# Explaining the decline in coronary heart disease mortality rates in the Slovak Republic between 1993-2008

**DOI:** 10.1371/journal.pone.0190090

**Published:** 2018-01-10

**Authors:** Marek Psota, Piotr Bandosz, Eva Gonçalvesová, Mária Avdičová, Mária Bucek Pšenková, Martin Studenčan, Jarmila Pekarčíková, Simon Capewell, Martin O'Flaherty

**Affiliations:** 1 Department of Public Health, Faculty of Health Sciences and Social Work, Trnava University in Trnava, Trnava, Slovak Republic; 2 PHARM-In, spol. s r.o., Bratislava, Slovak Republic; 3 Department of Public Health and Policy, Institute of Psychology, Health & Society, University of Liverpool, Liverpool, United Kingdom; 4 Department of Heart Failure and Transplantation, National Institute of Cardiovascular Diseases, Bratislava, Slovak Republic; 5 Regional Authority of Public Health in Banská Bystrica, Banská Bystrica, Slovak Republic; 6 Cardiac centre of Faculty Hospital J.A.Reiman, Prešov, Slovak Republic; University of Tampere, FINLAND

## Abstract

**Objective:**

Between the years 1993 and 2008, mortality rates from coronary heart disease (CHD) in the Slovak Republic have decreased by almost one quarter. However, this was a smaller decline than in neighbouring countries. The aim of this modelling study was therefore to quantify the contributions of risk factor changes and the use of evidence-based medical therapies to the CHD mortality decline between 1993 and 2008.

**Methods:**

We identified, obtained and scrutinised the data required for the model. These data detailed trends in the major population cardiovascular risk factors (smoking, blood pressure, total cholesterol, diabetes prevalence, body mass index (BMI) and physical activity levels), and also the uptake of all standard CHD treatments. The main data sources were official statistics (National Health Information Centre and Statistical Office of the Slovak Republic) and national representative studies (AUDIT, SLOVAKS, SLOVASeZ, CINDI, EHES, EHIS). The previously validated IMPACT policy model was then used to combine and integrate these data with effect sizes from published meta-analyses quantifying the effectiveness of specific evidence-based treatments, and population-wide changes in cardiovascular risk factors. Results were expressed as deaths prevented or postponed (DPPs) attributable to risk factor changes or treatments. Uncertainties were explored using sensitivity analyses.

**Results:**

Between 1993 and 2008 age-adjusted CHD mortality rates in the Slovak Republic (SR) decreased by 23% in men and 26% in women aged 25–74 years. This represented some 1820 fewer CHD deaths in 2008 than expected if mortality rates had not fallen. The IMPACT model explained 91% of this mortality decline. Approximately 50% of the decline was attributable to changes in acute phase and secondary prevention treatments, particularly acute and chronic treatments for heart failure (≈12%), acute coronary syndrome treatments (≈9%) and secondary prevention following AMI and revascularisation (≈8%). Changes in CHD risk factors explained approximately 41% of the total mortality decrease, mainly reflecting reductions in total serum cholesterol. However, other risk factors demonstrated adverse trends and thus generated approximately 740 additional deaths.

**Conclusion:**

Our analysis suggests that approximately half the CHD mortality fall recently observed in the SR may be attributable to the increased use of evidence-based treatments. However, the adverse trends observed in all the major cardiovascular risk factors (apart from total cholesterol) are deeply worrying. They highlight the need for more energetic population-wide prevention policies such as tobacco control, reducing salt and industrial trans fats content in processed food, clearer food labelling and regulated marketing of processed foods and sugary drinks.

## Introduction

Cardiovascular diseases are still the leading cause of death worldwide [1]. However, in recent decades, mortality rates from CHD have halved in the European Union and other high income countries [2–4]. Similar mortality declines have also been observed in Central Europe, particularly in the Czech Republic [5], Poland [6,7], Hungary [8,9] and Austria [10], although these did not start until the 1990s.

However, the CHD mortality decline in the Slovak Republic (SR) has been smaller and slower than many of its central European neighbours [4,11]. Between the years 1993–2008 age-adjusted CHD mortality rates in Slovakia decreased by just 23% in men (from 344.6 per 100 000 to 263.76 per 100 000) and by 26% in women (from 130.90 per 100 000 to 96.67 per 100 000) aged 25–74 years (Figure A and Table B in S1 Appendix). Explaining these trends is therefore important to help guide more effective public health actions.

The IMPACT model [12,13] has been used to explore CHD trends in more than 20 countries including Poland [7] and the Czech Republic [5]. The objective of current modelling study was to use the IMPACT model for explaining most of the fall in CHD mortality in Slovak population aged 25–74 between 1993 and 2008.

## Methods

To explain the changes in CHD mortality in Slovakia (ages 25–74) between 1993 and 2008 we used the IMPACT model, previously validated for example in the UK [14,15], Sweden [16], the Czech Republic [5], Poland [7] and elsewhere [13]. Descriptions of the model are detailed in S1 Appendix. In brief, the model aims to be comprehensive, incorporating all major cardiovascular risk factors, including systolic blood pressure (SBP), total cholesterol (TC), diabetes (DM), smoking, overweight and obesity (expressed as BMI) and physical inactivity (PA), plus all usual evidence based treatments for CHD and heart failure (HF). All available Slovak data sources were therefore systematically identified and critically reviewed as inputs in the Slovak IMPACT model. The analysis was confined to adults aged between 25–74 years. Age and sex specific mortality (ICD-10: I20 –I25 and I50, see S1 Appendix for more details) and demographic data were provided by the Statistical Office of the Slovak Republic. Age and sex specific numbers of patients with CHD as well as age and sex specific numbers of patients undergoing percutaneous transluminal coronary angioplasty (PTCA) and coronary artery bypass grafting (CABG), proportions of patients treated with evidence based treatments (statins, antihypertensive drugs, aspirin, β-blockers, thrombolysis) as well as the prevalence and mean values of selected risk factors were obtained either from routine statistics (National Health Information Centre) or from cross-sectional national and local studies; country representative surveys (CINDI [17], EHES [17], EHIS [18], AUDIT [19], SLOVAKS [20], SLOVASeZ [21], see S1 Appendix for more details on these surveys) and other modelling studies [22]. Expert opinions were also elicited where objective data were deficient. All assumptions and estimations are detailed in S1 Appendix.

### Estimating the number of deaths prevented or postponed

In order to estimate the number of deaths prevented or postponed over the period of interest, we subtracted the number of deaths from CHD expected in 2008 if the 1993 mortality rates had persisted, from the number of deaths actually observed in 2008 (see Example A in S1 Appendix). We then used the IMPACT methodology to estimate the proportions of that total number of DPPs that could be attributed to the use of treatments and to changes in cardiovascular risk factors. All estimates were stratified by age and gender.

### Benefits from treatments

Data on the clinical effectiveness of each intervention and therapy were based on the most recent meta-analyses and large randomized clinical trials (see Table D in S1 Appendix).

The number of DPPs as a result of each individual intervention in each group of CHD patients in the year 2008 was then calculated by multiplying the number of patients in each diagnostic group by their baseline case-fatality rate over 1 year, the proportion of these patients receiving a specific treatment, and the relative reduction in one–year case-fatality by the treatment (see Example B in S1 Appendix).

In 1993, several evidence-based treatments were not used in Slovakia (e.g. statins, or primary angioplasty in acute myocardial infarction). However, in some cases the use of some drugs or procedures in 1993 was not negligible (e.g. antihypertensive treatment or aspirin in acute myocardial infarction). In such cases, in order to obtain the net benefit, the number of DPPs as a result of the therapy as used in 1993 was calculated and subtracted from the number calculated for 2008. Only net DPPs are shown in results tables.

Compliance was assumed to be 100% among hospital patients, 70% among symptomatic community patients and 50% among asymptomatic community patients [23]. To avoid double counting of patients, it was necessary to identify potential overlaps between different groups of patients. Assumptions concerning these overlaps are presented in Table I in S1 Appendix. To quantify the relative reduction in case-fatality rate for individual patients receiving multiple treatments, we used the conventional Mant and Hicks cumulative relative benefit approach [24].

### Risk factors changes and their impact on CHD mortality

Two approaches were used to calculate the numbers of DPPs as a result of changes in risk factors. We used a regression approach for risk factors expressed as continuous data, i.e. SBP, TC concentration and BMI. The number of DPPs due to change in risk factor was then calculated as the product of the number of CHD deaths expected in the final year (2008) if the baseline year (1993) mortality persisted, the change in risk factor level and the regression coefficient (published in literature) quantifying the change in CHD mortality per unit of absolute change in that risk factor (see Example C in S1 Appendix).

The effect of changes in the prevalence of smoking, DM and PA was estimated using a population attributable risk fraction approach. In this method, the number of expected CHD deaths in the final year if the baseline year mortality persisted was multiplied by the difference between the population-attributable risk fraction in 1993 and that in 2008 (see Example D in S1 Appendix).

We assumed that there was no further synergy between the treatment and risk factor sections of the model, or between the major risk factors because the regression coefficients and relative risks for each risk factor were each independent. DPPs as a result of risk factor changes were then systematically quantified for each patient group. Lag times between risk factor rate change and event rate change were not explicitly modelled. We assumed, as in other countries, that any time lag would be relatively unimportant over a period of sixteen years (1993–2008) [25]. All results were rounded to the nearest multiple of five.

### Model validation: Comparison of estimated with observed mortality changes

The model produces estimates of the total number of CHD DPPs attributable to each treatment and changes in specific risk factors. These estimates were then added up and compared with the observed changes in mortality for men and women in each specific age group. Any shortfall in the overall model estimate was then presumed to reflect imprecision in model or other unmeasured risk factors.

### Sensitivity analyses

All the above assumptions were tested in a multi-way sensitivity analysis using the analysis of extremes methods [26]. For each model parameter, a lower and upper value was assigned using 95% confidence intervals where available, or otherwise using ± 20% values (for patient numbers, treatment uptake, and compliance). More detailed information concerning the methodology is provided in Example F in S1 Appendix.

## Results

### Deaths prevented or postponed

The observed decline in CHD mortality rates in the Slovak population aged 25–74 years since 1993, resulted in some 1820 fewer CHD deaths in 2008 (1205 in men and 615 in women).

### Treatments benefits

All treatments combined accounted for approximately 915 fewer deaths (some 520 in men and 395 in women aged 25–74 years), representing about 50% of the overall mortality decrease. Estimated numbers of DPPs in men and women resulting from medical and surgical treatments in 2008 are summarised in Table 1 and detailed in Table K in S1 Appendix. The largest mortality reductions came from heart failure treatments in hospital and in the community which resulted in approximately 210 fewer deaths in 2008 (12% of the observed mortality reduction). Initial treatments for acute myocardial infarction or unstable angina generated about 165 fewer deaths, 9% of the observed fall. Secondary prevention therapies after myocardial infarction or revascularization explained 150 (8%) fewer deaths. Chronic angina treatments explained 80 (4%) fewer deaths. Hypertension treatments explained approximately 145 (8%) DPPs and statins for primary prevention explained 165 (9%) DPPs. The contribution of different treatment methods was generally consistent when compared men and women with the exception of statins for hypercholesterolemia in women where this treatment explained 19% DPPs.

**Table 1:** Effects of treatments methods to the CHD mortality decrease between the years 1993–2008 in the population of 25–74 years old Slovaks.

	Total population		Men		Woman	
	DPPs Best estimate and range*	% of total fall in mortality (range)	DPPs Best estimate and range*	% of total fall in mortality in men (range)	DPPs Best estimate and range*	% of total fall in mortality in women (range)
Initial treatments of AMI	75 (40–140)	4.1 (2.1–7.6)	60 (30–105)	4.8 (2.5–8.8)	20 (10–30)	2.9 (1.3–5.3)
Unstable angina	90 (40–180)	4.9 (2.0–9.8)	55 (20–110)	4.5 (1.8–9.0)	35 (15–70)	5.8 (2.4–11.5)
Secondary prevention following AMI	130 (35–365)	7.0 (2.0–20.0)	85 (25–245)	7.3 (2.1–20.5)	40 (10–120)	6.6 (1.8–19.2)
Secondary prevention following CABG and PTCA	20 (5–55)	1.2 (0.4–3.0)	15 (5–40)	1.3 (0.4–3.2)	5 (0–15)	1.0 (0.3–2.5)
Stable angina	80 (25–205)	4.4 (1.5–11.2)	40 (15–95)	3.3 (1.2–8.0)	40 (10–110)	6.5 (2.0–17.5)
Hospital heart failure	100 (30–275)	5.5 (1.6–15.1)	60 (20–170)	5.2 (1.5–14.2)	40 (10–105)	6.2 (1.8–17.0)
Community heart failure	110 (35–285)	6.1 (1.9–15.6)	70 (25–185)	5.9 (1.9–15.2)	40 (10–100)	6.4 (2.0–16.5)
Statins (primary prevention)	165 (55–405)	9.0 (2.9–22.3)	45 (15–110)	3.7 (1.2–9.3)	120 (40–295)	19.2 (6.3–47.8)
Antihypertensives (primary prevention)	145 (25–205)	8.0 (1.3–11.3)	85 (15–125)	7.2 (1.2–10.5)	60 (10–80)	9.4 (1.6–12.8)
**Total treatments**	**915**	**50.2**	**515**	**43.2**	**400**	**64.0**

* The absolute numbers of DPPs were rounded to the nearest multiple of 5 (e.g. 6 became 5), therefore small inaccuracies may occur

### Risk factor changes

Estimated effects of risk factors are summarised in Table 2. Most of them showed adverse trends, generating additional deaths.

**Table 2:** Effects of risk factors changes to the CHD mortality decrease between the years 1993–2008 in the population of 25–74 years old Slovaks.

	Prevalence/average ^a^		Change	Deaths prevented or postponed			Deaths prevented or postponed (%)		
	1993	2008	Absolute	Best	Min.	Max.	Best	Min.	Max.
**Smoking**	**0.23**	**0.20**	**0.03**	**-310**	**-370**	**-245**	***-17***	***-20***	***-14***
Males	0.29	0.25	0.04	-125	-150	-100	*-7*	*-8*	*-6*
Females	0.17	0.14	0.03	-185	-220	-145	*-10*	*-12*	*-8*
**Systolic blood pressure**	**132.0**	**131.9**	**0.08**	**-220**	**-600**	**195**	***-12***	***-33***	***11***
Males	133.8	136.6	-2.81	-510	-750	-285	*-28*	*-41*	*-16*
Females	130.3	127.5	2.82	290	150	475	*16*	*8*	*26*
**Cholesterol**	**5.78**	**5.19**	**0.60**	**1490**	**380**	**2335**	***82***	***21***	***128***
Males	5.66	5.13	0.53	1120	485	1640	*61*	*27*	*90*
Females	5.89	5.24	0.66	370	-105	695	*20*	*-6*	*38*
**Physical inactivity**	**0.28**	**0.29**	**-0.003**	**-90**	**-105**	**-70**	***-5***	***-6***	***-4***
Males	0.22	0.27	-0.05	-80	-100	-65	*-4*.*5*	*-5*.*4*	*-3*.*6*
Females	0.34	0.30	0.04	-5	-10	-5	*-0*.*4*	*-0*.*4*	*-0*.*3*
**Body Mass Index**	**26.34**	**26.60**	**-0.26**	**-70**	**-110**	**-40**	***-4***	***-6***	***-2***
Males	26.56	27.21	-0.65	-60	-90	-30	*-3*	*-5*	*-2*
Females	26.12	26.02	0.10	-15	-20	-5	*-0*.*7*	*-1*.*1*	*-0*.*4*
**Diabetes**	**0.09**	**0.10**	**-0.01**	**-55**	**-65**	**-45**	***-3***	***-4***	***-2***
Males	0.09	0.10	-0.01	-45	-55	-35	*-2*	*-3*	*-2*
Females	0.090	0.093	-0.003	-10	-15	-10	*-0*.*6*	*-0*.*7*	*-0*.*5*
**Total risk factors**				**745**	**-**	**-**	***41%***	***-***	***-***

^a^ weighted average of all age groups and both sexes

The absolute numbers of DPPs were rounded to the nearest multiple of 5 (e.g. 6 became 5), therefore small inaccuracies may occur.

Negative values denote additional deaths

Despite the overall prevalence of smoking decreased by 3% over the period, persistently high smoking prevalence in older groups resulted in 310 additional deaths.

In the observed period SBP trends increased in men but decreased slightly in women. The adverse trends resulted in 220 additional deaths, after subtracting the effect of antihypertensive treatments.

Adverse trends in physical inactivity, BMI and diabetes prevalence resulted in approximately 215 additional deaths (90, 70 and 55 additional deaths respectively).

The only risk factor showing favourable trends was total cholesterol. A decrease of total cholesterol of 0.6 mmol/l resulted in approximately 1490 fewer deaths.

The net result of these contrasting trends was that 745 deaths were postponed or prevented by risk factor changes (41% of the observed fall in mortality), thanks solely to the modest reductions in population cholesterol levels (and not including reductions attributable to statins).

### Sensitivity analysis and model validation

Our results remained reasonably stable in the sensitivity analysis (Figure C in S1 Appendix). The model explained 1660 of the estimated 1820 DPPs with an overall model fit of 91%. Fit in older age groups was not so good (Figure D in S1 Appendix).

## Discussion

Age-adjusted CHD mortality rates in the Slovak adult population decreased by a quarter between 1993 and 2008. Our model suggests that approximately half of that CHD mortality decline might be attributed to the increased use of evidence-based treatments, and approximately 40% might be explained by improvements in risk factors, principally total cholesterol. This 40% is smaller than the contribution seen in neighbouring countries like in Poland (54%) [7] and the Czech Republic (52%) [5].

The most important driver of the decrease in CHD mortality in the SR was the contribution of evidence-based treatments, mainly acute and chronic HF and treatments of AMI. This perhaps is not surprising. Since 1993, the number of invasive cardiology centres has doubled from three to six in the country and the number of PTCA increased exponentially from approximately 125 in 1993 to 5575 in 2008, many being performed in the ACS setting. We also observed substantial improvements in the uptake of key pharmacological treatments such as ACE inhibitors, β-blockers and statins in all disease groups.

Primary prevention treatments also helped; and about 8% of total DPPs could be attributed to increased use of antihypertensive therapies and 9% to statins. We observed considerable differences in contribution of statins as primary prevention between men and women. While statins in this setting explained approximately 4% DPPs in men, they apparently explained 19% DPPs in women. This discrepancy might partly reflect a frustrating lack of local data on statin uptake as primary prevention as well as on the number of eligible patients. The best available data from CINDI and EHES surveys were both limited to ages less than 65. We therefore had to estimate inputs for the group aged 65–74 years (see S1 Appendix for more details).

Treatment contributions alone were not powerful enough to achieve faster rates in mortality decline, particularly given the generally adverse trends in risk factors which resulted in additional deaths.

Thus between 1993 and 2008, although systolic blood pressure decreased in women, it increased in men generating over 200 additional deaths. A similar phenomenon was also observed in the Polish population [7]. The reason of these important and worrying sex differences in blood pressure trends remains unclear, and more research is clearly needed.

Despite the decreasing overall prevalence of smoking, the effect of smoking in the model is negative (310 additional deaths), mainly reflecting the increasing prevalence in older men and women. Similar trend but only in women was observed also in neighbouring Czech [5] and Polish [7] populations. This worrying phenomenon clearly needs further analysis.

Decreasing physical activity especially in males generated 90 additional deaths. Similar adverse trends have also been observed in Ireland [27] and the West Bank [28]. Similarly, increases in diabetes prevalence and BMI generated almost 130 additional deaths.

Only total cholesterol showed significant improvements (a 0.53 mmol/l reduction in men and 0.66 mmol/l in women). This contributed almost 1500 fewer deaths, essentially all the fewer deaths attributable to risk factors improvements. This substantial contribution is consistent with trends in the Czech Republic [5], Poland [7] and many other Western European countries [16,27,29–31]. In Central European countries, favourable changes in diet after the fall of the communist economy were observed. The free market provided relatively cheap vegetable oils, fruit and vegetables in Poland after 1990. The polyunsaturated/saturate ratio improved substantially and CHD rates fell by a quarter within five years. Similar trends were reported in East Germany, Hungary and the Czech Republic [6,32,33]. Likewise in Slovakia, the ratio of animal to vegetable fats supplies decreased from approximately 2 to approximately 1.3 over the study period [34]. The consumption of beef, pork, pork fat and butter decreased while consumption of poultry and fish, vegetable oils, fruits and nuts all increased [35]. Population wide changes in diet might thus be responsible for the substantial declines in cholesterol levels and corresponding mortality falls in the region.

### Strengths and limitations

The IMPACT model is comprehensive, and takes into account all known important downstream risk factors and all standard treatments. The model has been previously validated in many Western countries and its results are consistent with other analyses performed in the same settings. However, several limitations should also be considered.

First, the Slovak IMPACT model was able to explain 91% of estimated DPPs but the fit within specific age groups was less perfect, much as in some other studies [28]. The remaining 9% unexplained by the model may reflect imprecisions in factor measurement, and also failure to include all the possible CHD risk factors, such as socioeconomics or psychosocial measures, environmental factors or fruit and vegetable consumption.

Second, quality issues with mortality data and coding quality might be an issue in Slovakia [36,37], affecting precise number of deaths explained by the model. However, Mathers et al. [38] listed Slovakia among the countries with mortality data of high quality, adequate for public health analysis. We also assumed a minimal lag time between changes in risk factors and changes in mortality, perhaps reasonably [39].

Although data from the CINDI studies comes from regular surveys in single one district in the middle of the country that might be cautiously generalizable to the wider population [17]. In many cases data even did not exist. This can happen even in countries with far more developed surveillance systems. We therefore needed to make appropriate assumptions when primary data was not available. In line with modelling best practice, we have detailed and summarized all our estimations in S1 Appendix, and tested these in the sensitivity analysis. Slovakia clearly needs a monitoring system of non-communicable disease morbidity and determinants to better inform public health policy. Sadly, since 2012 when the last EHES study was conducted, to our knowledge there has only been one European Health Interview Survey (EHIS 2014) and that does not include objective measurements such as SBP or TC [40].

Third, the model used data on treatment efficacy from randomized controlled trials that may overestimate benefits in the wider population. Few specific Slovak data were available on case-fatality rates, so these were based on data from the United Kingdom and US. Residual double counting of some individual patients also remains possible.

Modern cardiologic treatments have made a significant contribution to the modest decline in CHD mortality, but more can be done. Effective, population-wide policies focused on tobacco control, reducing of salt and trans fats content in industrial food, appropriate and understandable food labelling and advertisement control may be effective in fighting CHD. They are also cost-saving and equitable, and can deliver results surprisingly rapidly [39,41]. Downstream interventions focussed on individuals appear less effective [42].

In conclusion, this modelling study is to our knowledge the first attempt to conduct a comprehensive analysis of CHD mortality trends integrating available data about risk factors and treatments uptake in Slovakia, and contributes to understand the complex reasons behind the decline of CHD mortality in Central Europe. The adverse trends observed in most cardiovascular risk factors are deeply worrying, and emphasise the need for more energetic population-wide prevention policies.

## Supporting information

S1 Appendix Appendix for the Slovak IMPACT model, 1993–2008. (DOCX)

## References

[1] WHO. The top 10 causes of death. Fact sheet no 310, updated January 2017. Available from: http://www.who.int/mediacentre/factsheets/fs310/en/

[2] Ford E, Ajani U, Croft J, Critchley J, Labarthe D, Kottke T et al Explaining the decrease in U.S. deaths from coronary disease, 1980–2000. N Engl J Med. 2007; 356: 2388–2398. doi: 10.1056/NEJMsa053935 17554120 10.1056/NEJMsa053935

[3] Wijeysundera HC, Machado M, Farahati F, Wang XS, Witteman W, van der Velde G et al Temporal Trends in Risk Factors and Treatment Uptake With Coronary Heart Disease Mortality, 1994–2005. JAMA. 2010; 303: 1841–1847. doi: 10.1001/jama.2010.580 20460623 10.1001/jama.2010.580

[4] Nichols M, Townsend N, Scarborough P, Rayner M. Trends in age-specific coronary heart disease mortality in the European Union over three decades: 1980–2009. Eur Heart J. 2013; 34: 3017–3027. doi: 10.1093/eurheartj/eht159 23801825 10.1093/eurheartj/eht159 PMC3796269

[5] Bruthans J, Cífková R, Lánská V, O'Flaherty M, Critchley JA, Holub J et al Explaining the decline in coronary heart disease mortality in the Czech Republic between 1985 and 2007. Eur J Prev Cardiol. 2014; 21: 829–839. doi: 10.1177/2047487312469476 23180867 10.1177/2047487312469476

[6] Zatonski WA, McMichael AJ, Powles JW. Ecological study of reasons for sharp decline in mortality from ischaemic heart disease in Poland since 1991. BMJ. 1998; 316: 1047–1051. 9552904 10.1136/bmj.316.7137.1047 PMC28506

[7] Bandosz P, O'Flaherty M, Drygas W, Rutkowski M, Koziarek J, Wyrzykowski B et al Decline in mortality from coronary heart disease in Poland after socioeconomic transformation: modelling study. BMJ. 2012; 344: d8136–d8136. doi: 10.1136/bmj.d8136 22279114 10.1136/bmj.d8136 PMC3266431

[8] Balogh S, Papp R, Jozan P, Csaszar A. Continued improvement of cardiovascular mortality in Hungary—impact of increased cardio-metabolic prescriptions. BMC Public Health. 2010; 10: 422 doi: 10.1186/1471-2458-10-422 20633257 10.1186/1471-2458-10-422 PMC2919475

[9] Bertuccio P, Levi F, Lucchini F, Chatenoud L, Bosetti C, Negri E et al Coronary heart disease and cerebrovascular disease mortality in young adults: recent trends in Europe. Eur J Cardiovasc Prev Rehabil. 2011; 18: 627–634. doi: 10.1177/1741826710389393 21521726 10.1177/1741826710389393

[10] WHO. European health for all database (HFA-DB) WHO/Europe. Updated July 2017. [cited 2017 Dec 30]. Available from: http://data.euro.who.int/hfadb/

[11] Psota M, Pekarcikova J, O'Mullane M, Rusnak M. Trends in age-adjusted coronary heart disease mortality rates in Slovakia between 1993 and 2009. Cent Eur J Publ Health. 2013; 21: 72–79. 10.21101/cejph.a3779 24053062

[12] Capewell S, Morrison C, McMurray J. Contribution of modern cardiovascular treatment and risk factor changes to the decline in coronary heart disease mortality in Scotland between 1975 and 1994. Heart. 1999; 81: 380–386. 10092564 10.1136/hrt.81.4.380 PMC1729021

[13] Psota M, Capewell S, O'Flaherty M, Goncalvesova E. Príčiny zmien v úmrtnosti na ischemickú chorobu srdca podľa modelu IMPACT: systematický prehľad / The causes of changes in coronary heart disease mortality rates using the IMPACT model: a systematic review. Cardiology Lett. 2013; 22: 449–458.

[14] Bajekal M, Scholes S, Love H, Hawkins N, O'Flaherty M, Raine R et al Analysing Recent Socioeconomic Trends in Coronary Heart Disease Mortality in England, 2000–2007: A Population Modelling Study. PLoS Medicine. 2012; 9: 1237–1237. 10.1371/journal.pmed.1001237 PMC3373639 22719232

[15] Unal B, Critchley JA, Capewell S. Modelling the decline in coronary heart disease deaths in England and Wales, 1981–2000: comparing contributions from primary prevention and secondary prevention. BMJ. 2005; 331: 614–617. doi: 10.1136/bmj.38561.633345.8F 16107431 10.1136/bmj.38561.633345.8F PMC1215556

[16] Bjorck L, Rosengren A, Bennett K, Lappas G, Capewell S. Modelling the decreasing coronary heart disease mortality in Sweden between 1986 and 2002. Eur Heart J. 2009; 30: 1046–1056. doi: 10.1093/eurheartj/ehn554 19141562 10.1093/eurheartj/ehn554

[17] Avdičová M, Francisciová K, Ďateľová M. Monitorovanie rizikových faktorov chronických chorôb v SR Banská Bystrica: Regionálny úrad verejného zdravotníctva so sídlom v Banskej Bystrici; 2012.

[18] Gerhardtová A. EHIS 2009 –Európske zisťovanie o zdraví 2009 Štatistický úrad Slovenskej republiky; 2011.

[19] Cagáň S, Trnovec T, Riečanský I, Jurkovičová O, Wimmerová S, Besedová I. The AUDIT Project: Factors affecting thrombolytic therapy in 3,123 patients with acute myocardial infarction. J Clin Basic Cardiol. 2002; 5: 183–188.

[20] Kovář F, Studenčan M, Hricák V, Kurray P, Murín J, Kamenský G et al Management of patients with acute coronary syndrome without ST-segment elevation. Analysis of the SLOVAKS registry during 2008 year. Manažment pacientov s akútnym koronárnym syndrómom bez elevácií segmentov ST. Analýza údajov registra SLOVAKS z roku 2008. Cardiology sk. 2010; 19: 181–191.

[21] Gonçalvesová E, Varga I, Lesný P, Líška B, Luknár M, Solík P. Charakteristiky a osud pacientov s akútnym srdcovým zlyhávaním v aktuálnej klinickej praxi. Vnitř lék. 2010; 56: 845–853. 20845617

[22] Danaei G, Finucane M, Lu Y, Singh G, Cowan M, Paciorek C et al National, regional, and global trends in fasting plasma glucose and diabetes prevalence since 1980: systematic analysis of health examination surveys and epidemiological studies with 370 country-years and 2.7 million participants. Lancet. 2011; 378: 31–40. doi: 10.1016/S0140-6736(11)60679-X 21705069 10.1016/S0140-6736(11)60679-X

[23] Nichol M, Venturini F, Sung J. A critical evaluation of the methodology of the literature on medication compliance. Ann Pharmacother. 1999; 33: 531–540. doi: 10.1345/aph.18233 10369613 10.1345/aph.18233

[24] Mant J, Hicks N. Detecting differences in quality of care: the sensitivity of measures of process and outcome in treating acute myocardial infarction. BMJ. 1995; 311: 793–796. 7580444 10.1136/bmj.311.7008.793 PMC2550793

[25] Capewell S, O'Flaherty M. Mortality falls can rapidly follow population-wide risk factor changes. Lancet. 2011; 378: 752–753. doi: 10.1016/S0140-6736(10)62302-1 21414659 10.1016/S0140-6736(10)62302-1

[26] Briggs A, Sculpher MJ, Buxton MJ. Uncertainty in the economic evaluation of health care technologies: the role of sensitive analysis. Health Economics. 1994; 3: 95–104. 8044216 10.1002/hec.4730030206

[27] Bennett K, Kabir Z, Unal B, Shelley E, Critchley J, Perry I et al Explaining the recent decrease in coronary heart disease mortality rates in Ireland, 1985–2000. J Epidemiol Community Health. 2006; 60: 322–327. doi: 10.1136/jech.2005.038638 16537349 10.1136/jech.2005.038638 PMC2566167

[28] Abu-Rmeileh NM, Shoaibi A, O'Flaherty M, Capewell S, Husseini A. Analysing falls in coronary heart disease mortality in the West Bank between 1998 and 2009. BMJ Open. 2012; 2 doi: 10.1136/bmjopen-2012-001061 22923626 10.1136/bmjopen-2012-001061 PMC3432845

[29] Laatikainen T, Critchley J, Vartiainen E, Salomaa V, Ketonen M, Capewell S. Explaining the decline in coronary heart disease mortality in Finland between 1982 and 1997. Am J Epidemiol. 2005; 162: 764–773. doi: 10.1093/aje/kwi274 16150890 10.1093/aje/kwi274

[30] Aspelund T, Gudnason V, Magnusdottir BT, Andersen K, Sigurdsson G, Thorsson B et al Analysing the Large Decline in Coronary Heart Disease Mortality in the Icelandic Population Aged 25–74 between the Years 1981 and 2006. PLoS One. 2010; 5: e13957 doi: 10.1371/journal.pone.0013957 21103050 10.1371/journal.pone.0013957 PMC2980472

[31] Flores-Mateo G, Grau M, O'Flaherty M, Ramos R, Elosua R, Violan-Fors C et al Analyzing the Coronary Heart Disease Mortality Decline in a Mediterranean Population: Spain 1988–2005. Rev Esp Cardiol. 2011; 64: 988–996. doi: 10.1016/j.recesp.2011.05.033 21962958 10.1016/j.recesp.2011.05.033

[32] Zatonski W, Willett W. Changes in dietary fat and declining coronary heart disease in Poland: population based study. BMJ. 2005; 331: 187–188. doi: 10.1136/bmj.331.7510.187 16037448 10.1136/bmj.331.7510.187 PMC1179759

[33] Capewell S, Ford E. Why have total cholesterol levels declined in most developed countries? BMC Public Health. 2011; 11: 641 doi: 10.1186/1471-2458-11-641 21834954 10.1186/1471-2458-11-641 PMC3199603

[34] FAOSTAT. [cited 2017 Dec 30]. Available from: http://faostat3.fao.org/home/E

[35] Statistical Office of the Slovak Republic. SLOVSTAT. [cited 2017 Dec 30] Available from: http://www.statistics.sk/pls/elisw/vbd

[36] Baráková A, Diba S, Hlava P. The structure of deaths causes in Slovakia is confused. Cardiology Lett. Suppl 2012: 9S.

[37] Baráková A, Brašeňová D, Dovičovič R, Hajnaliová J, Hlava P. Objektivizácia príčin smrti a relevantnosti štatistických údajov v listoch o prehliadke mŕtveho Informačno-analytická štúdia. Bratislava: Národné Centrum Zdravotníckych Informácií; 2011.

[38] Mathers CD, Fat DM, Inoue M, Rao C, Lopez AD. Counting the dead and what they died from: An assessment of the global status of cause of death data. Bull World Health Organ. 2005; 83: 171–177. 15798840 PMC2624200

[39] Capewell S, O'Flaherty M. Can dietary changes rapidly decrease cardiovascular mortality rates? Euro Heart J. 2011; 32: 1187–1189. 10.1093/eurheartj/ehr049 21367835

[40] Statistical Office of the Slovak Republic. Pohľad na zdravotný stav obyvateľstva SR a jeho determinanty (výsledky EHIS 2014). Bratislava; 2015.

[41] NICE. Prevention of cardiovascular disease. London; 2010.

[42] Ebrahim S, Taylor F, Ward K, Beswick A, Burke M, Davey Smith G. Multiple risk factor interventions for primary prevention of coronary heart disease. Cochrane Database Syst Rev. 2011; 1: 1561 doi: 10.1002/14651858.CD001561.pub3 21249647 10.1002/14651858.CD001561.pub3 PMC11729147

