# Supplementary material for: Explaining the decline in coronary heart disease mortality rates in the Slovak Republic between 1993-2008
Source: PLoS One. 2018 Jan 10;13(1):e0190090. doi: 10.1371/journal.pone.0190090 (PMC5761866; doi:10.1371/journal.pone.0190090)
Supplement: S1 Appendix — (DOCX) [file pone.0190090.s001.docx]

|  |
| --- |
| **Appendix for the Slovak IMPACT model** |
| Explaining the decline in coronary heart disease mortality rates in the Slovak Republic between 1993-2008 |
|  |

**Marek Psota^1,2*^**, **Piotr Bandosz^3^**, **Eva Gonçalvesová^4^**, **Mária Avdičová^5^**, **Mária Bucek Pšenková^2^**, **Martin Studenčan^6^**, **Jarmila Pekarčíková^1^**, **Simon Capewell^3^**, **Martin O'Flaherty^3^**

^1^Department of Public Health, Faculty of Health Sciences and Social Work, Trnava University in Trnava, Trnava, Slovak Republic

^2^PHARM-In, spol. s r.o., Bratislava, Slovak Republic

^3^Department of Public Health and Policy, Institute of Psychology, Health & Society, University of Liverpool, Liverpool, United Kingdom

^4^Department of Heart Failure and Transplantation, National Institute of Cardiovascular Diseases, Bratislava, Slovak Republic

^5^Regional Authority of Public Health in Banská Bystrica, Banská Bystrica, Slovak Republic

^6^Cardiac centre of Faculty Hospital J.A.Reiman, Prešov, Slovak Republic

*Corresponding author

mpsota@gmail.com (MP)

**CONTENTS**

[**1.** **INTRODUCTION AND METHODS** 4](#_Toc502065304)

[1.1 Changes in mortality rates from CHD in the Slovak Republic from 1993 to 2008 5](#_Toc502065305)

[1.2 Heart failure group/deaths 7](#_Toc502065306)

[1.3 Expected and observed number of deaths from CHD 8](#_Toc502065307)

[1.4 Calculation of benefits from treatments 8](#_Toc502065313)

[1.5 Calculation of influence from risk factors 12](#_Toc502065314)

[1.6 Other methodological considerations 16](#_Toc502065318)

[1.7 Sensitivity analysis 17](#_Toc502065322)

[1.8 Treatments effects from the initial year 18](#_Toc502065323)

[**2.** **LIST OF ASSUMPTIONS** 24](#_Toc502065325)

[**3.** **REFERENCES** 27](#_Toc502065327)

[**Table A** Model populations and data sources used in the Slovak IMPACT model 5](#_Toc502067422)

[**Table B** CHD mortality rates in Slovakia between 1993-2008, population ages 25-74 years 7](#_Toc502067423)

[**Table C** CHD (plus 50% of Heart failure deaths) mortality rates in Slovakia between 1993-2008, population ages 25-74 years 7](#_Toc502067424)

[**Table D** Clinical efficacy of interventions: relative risk reductions obtained from meta-analyses, and randomized controlled trials 9](#_Toc502067425)

[**Table E** Treatments uptake (weighted average of all age groups) and data sources 10](#_Toc502067426)

[**Table F** Risk factors data sources 12](#_Toc502067427)

[**Table G** Estimated β coefficients from multiple regression analyses for the relationship between absolute changes in population mean risk factors and percentage changes in coronary heart disease mortality for men and women, stratified by age. Data sources, values and comments. 13](#_Toc502067428)

[**Table H** Relative risks used in the Slovak IMPACT model for smoking and diabetes for CHD mortality 15](#_Toc502067429)

[**Table I** Overlap adjustments in the Slovak IMPACT model 17](#_Toc502067430)

[**Table J** Case fatality rates for each patient group 19](#_Toc502067431)

[**Table K** Effects of treatments methods to the CHD mortality decrease between the years 1993-2008 in the population of 25-74 years old Slovaks (extended) 20](#_Toc502067432)

[**Figure A** Age-adjusted mortality from CHD in Slovak population aged 25-74 years between years 1993 – 2012 (both sexes). 6](#_Toc502067532)

[**Figure B** Age-adjusted mortality from CHD (plus 50% of Heart failure deaths) in Slovak population aged 25-74 years between years 1993 – 2012 (both sexes). 7](#_Toc502067533)

[**Figure C** Contribution of treatments and risk factors changes to the CHD mortality decrease in the population of 25-74 years old Slovaks. Diamonds represent the best estimate; lines are extremes of sensitivity analysis. 23](#_Toc502067534)

[**Figure D** Comparison of observed and explained number of DPPs according to the Slovak IMPACT model. 23](#_Toc502067535)

[**Example A** Calculation of DPPs in one age group. 8](#_Toc502067597)

[**Example B** Calculation of DPPs attributable to evidence based treatment 12](#_Toc502067598)

[**Example C** Calculation of DPPs attributable to a risk factor change using regression approach 13](#_Toc502067599)

[**Example D** Calculation of DPPs attributable to a risk factor change using PARF method 15](#_Toc502067600)

[**Example E** Mant and Hicks approach example 16](#_Toc502067601)

[**Example F** Sensitivity analysis example 17](#_Toc502067602)

# List of abbreviations

| **≈** | Almost equal |
| --- | --- |
| **AUDIT** | Audit of diagnostic and therapeutic management in patients with acute coronary syndromes |
| **CINDI** | Countrywide Integrated Non-communicable Disease Intervention Programme 1993 - 2008 |
| **EHIS** | European Health Interview Survey 2009 |
| **EHES** | European Health Examination Survey 2012 |
| **GBMD** | Global Burden of Metabolic Diseases |
| **NCZI** | National Health Information Centre, Slovak Republic |
| **NÚSCH** | National Institute for Heart Diseases, Bratislava, Slovak Republic |
| **SLOVAKS** | Slovak Registry of Acute Coronary Syndromes |
| **SLOVASeZ** | Slovak Registry of Acute Heart Failure |
| **ŠÚSR** | Statistical Office of the Slovak Republic |

# A brief description of studies and surveys used in the Slovak IMPACT model

| **AUDIT 1997-1998^1-3^** | Study of the Ministry of Health of SR "Audit of diagnostic and therapeutic management in patients with acute coronary syndromes (AUDIT). Pharmacotherapy and other recommended measures at discharge were analysed in 2527 patients discharged with the diagnosis of first or repeated myocardial infarction. Prospective multicentre study. |
| --- | --- |
| **SLOVAKS 2008^4^** | First attempt to establish the Slovak Acute coronary syndrome Registry. A cooperation of Slovak Society of Cardiology and National Health Information Centre. Study period 2007-2008, 10 643 ACS (4072 STEMI; 5282 NSTEMI/UAP). |
| **SLOVAKS- 2 (2011)^5^** | Change in the methodology. The cross-sectional study, period only two months (august, september 2011). 69 Slovak hospitals (85% of all). 1580 ACS (484 STEMI). |
| **SLOVASeZ 2009^6^** | National multicentre prospective study conducted between 1^st^ May and 31^st^ July 2009. The observed population were patients with acute heart failure (n= 860). |
| **Unpublished study of SKS** | Study of Slovak Society of Cardiology. Questionnaires sent to physicians following up patients with chronic heart failure. |
| **CINDI 1993,2008**^7^ | A cross-sectional population study that was a part of CINDI programme. A model district representative for the whole country was selected – Banska Bystrica. Smoking prevalence, anthropometric measurements (BMI), blood pressure (2x), cholesterol, glucose, etc. – laboratory. |
| **EHIS 2009**^8^ | A population based representative survey coordinated by EUROSTAT (European Health Interview Survey). Sample size 4972 respondents. No anthropometric measurements. The source of diabetes prevalence in final year. EHIS 2009 is a first sample survey on population health in the Slovak Republic (SR) that was performed on the basis of harmonised Eurostat methodology. The Statistical Office of the Slovak Republic realised the survey within a European project of public health statistics. The data was collected face to face by PAPI method (paper and pencil interview) and self-administered questionnaire. Interviewer completed the paper questionnaire on the basis of respondent’s answers. During the interview, interviewer asked respondent to complete two self-completion forms. Respondent completed the self-completion forms by him/herself and put the completed questionnaires into envelope, sealed it and gave to interviewer. Respondent could ask interviewer for help in completion of the questionnaire. |
| **EHES 2011**^7^ | Nationwide population study (European Health Examination Survey) conducted in 2011. Methodology similar to the CINDI screenings. Information about uptake od statins as primary prevention used (decreased by 10% to reflect the difference between the time of conducting EHES and our final year) |

1. **INTRODUCTION AND METHODS**

This supplementary appendix document provides details about the methods that were used in creating the Slovak IMPACT model. This model examines the effects of changes in treatments and risk factors trends on changes in mortality from coronary heart disease (CHD) among Slovak adults aged 25–74 years.

IMPACT is a deterministic, cell-based policy model. It uses epidemiological information to estimate the contributions of population-level risk factor changes (impacting mainly on incidence) and changes in the uptake of evidence-based treatments (impacting mainly on case fatality) on mortality decline between two points in time (the start-year and the end-year). The primary outcome measure of the model is the deaths prevented or postponed (DPPs).

The starting point for the model is to calculate the ‘target’ number of deaths the model needs to explain. This target number is obtained by using death counts recorded in the official registration system to calculate the difference between the actual observed CHD deaths recorded in the end-year and the deaths expected in the end-year had the CHD mortality rates remained the same as in the start-year (i.e. simple direct standardisation).

The calculation of the modelled estimate of DPPs rests on utilising two well-studied relationships: firstly, that between risk factor change and the relative reduction in CHD mortality; secondly, that between treatment uptake and reductions in case-fatality in patients with a specific form of CHD.

The model applies the relative risk reduction quantified in previous randomised controlled trials and meta-analyses to estimate the mortality reduction attributable to:

a) temporal change in risk factor prevalence (in those without diagnosed CHD) to calculate the DPPs ‘explained’ by specific risk factor trends;

b) net change over the period in the uptake of specific treatments in patients with each specific form of CHD to estimate DPPs ‘explained’ owing to improved 1-year case fatality rates. Great care is taken to avoid double counting the same individuals.

The mortality benefits from the risk factor reduction in the population, and the treatment benefits in patient groups are then summed. Thus summing uses a cumulative approach (rather than an additive approach), in order to avoid double-counting of benefits in the same individual.

This mortality sum represents the deaths prevented or postponed (DPPs) ‘explained’ by the model.

At the end of the modelling process, the total DPPs ‘explained’ by the model is then compared with the observed fall in deaths (the ‘target’ to be explained).

**Model fit** is therefore calculated as the difference between the observed deaths and model DPPs, and expressed as the percentage explained. This measures the extent to which the model was successful in explaining the observed change in CHD mortality in the population.

A policy model like IMPACT thus stands in contrast to a typical multivariate regression model. A typical multivariate regression model represents a statistical approach to describing a single data-set, for instance generated by a single cohort or randomised controlled trial. In contrast, a policy model such as IMPACT seeks to integrate and synthesise best estimates from a variety of sources to reliably estimate the extent to which a range of factors, acting in combination, explain or predict an outcome. We did not obtain the parameters for this model by running regressions. Rather, the model incorporates the best coefficients from the largest meta-analysis or randomised controlled trials of the reduction in case fatality attributed to treatment or the independent effect sizes of a unit change in each risk factor on CHD mortality.

Earlier versions of the IMPACT mortality model have been previously applied to data from Europe, New Zealand, China, the United States and Canada (Ontario).^9^ This cell-based mortality model, developed in Microsoft Excel, has been described in detail online and elsewhere.^10-30^

## 1.1 Changes in mortality rates from CHD in the Slovak Republic from 1993 to 2008

The data sources used in examining the changes in cardiovascular mortality rates from 1993 to 2008 among the Slovak population aged 25–74 years are shown in Table A. Mortality rates from CHD were calculated using the underlying cause of death: the International Classification of Diseases ICD-9 codes 410–414 and ICD-10 codes I20–I25. As we were only interested in deaths from coronary artery disease, we only included heart failure deaths that were a result of ischemic cardiomyopathy (for details, see below and Table A).

Table A Model populations and data sources used in the Slovak IMPACT model

|  | **1993** | **2008** | **Comments** |
| --- | --- | --- | --- |
| **Population** | ŠÚSR | ŠÚSR | population at midyear |
| **Deaths by age and sex**  *(ICD 9 410-414, 428; ICD 10 I20-I25, I50)* | ŠÚSR | ŠÚSR | 50% of I50 deaths arbitrarily added |
| **Numbers of patients admitted yearly into hospitals** | | | |
| **Myocardial infarction**  **(410, I21,I22)** | Estimate | NCZI | Number of patients admitted into hospitals yearly without double counting due to transfers among departments and hospitals.  Data available between years 1995 – 2008. First two years estimated. |
| **Unstable angina (413,I20)** | Estimate | NCZI |  |
| **Heart failure (428, I50)^[[1]](#footnote-1)^** | Estimate | NCZI |  |
| **Numbers of patients treated yearly with** | | | |
| **CABG** | Estimate | Estimate | Based on data from NUSCH, NCZI. |
| **PTCA** | Estimate | Estimate | Based on data from NUSCH |
| **Numbers of patients in the community** | | | |
| **Post MI** | Estimate | NCZI | Estimated from numbers of individuals admitted to hospital (cumulative probability of surviving, last 10 years). |
| **Community Stable Angina** | Estimate | EHIS 2009 | Only people without myocardial infarction |
| **Heart failure^[[2]](#footnote-2)^** | Estimate | NCZI | - |
| **Hypertension (eligible for antihypertensive treatment as primary prevention)** | CINDI | CINDI | Estimated as prevalence of people with SBP ≥140 mmHg or DBP ≥90mmHg or reporting having hypertension and simultaneously not having CHD or stroke. |
| **Hypercholesterolemia**  **(eligible for statins treatment as primary prevention)** | CINDI | CINDI | Estimated as prevalence of people with total cholesterol ≥5 mmol/l or reporting having disorders of lipid metabolism and simultaneously not having CHD or stroke. |

Both, unadjusted and adjusted mortality rates were calculated. Age standardization was done using the direct method based on the European standard population.^31^ The observed fall in the age-adjusted CHD mortality is shown on the figure A and in table B.


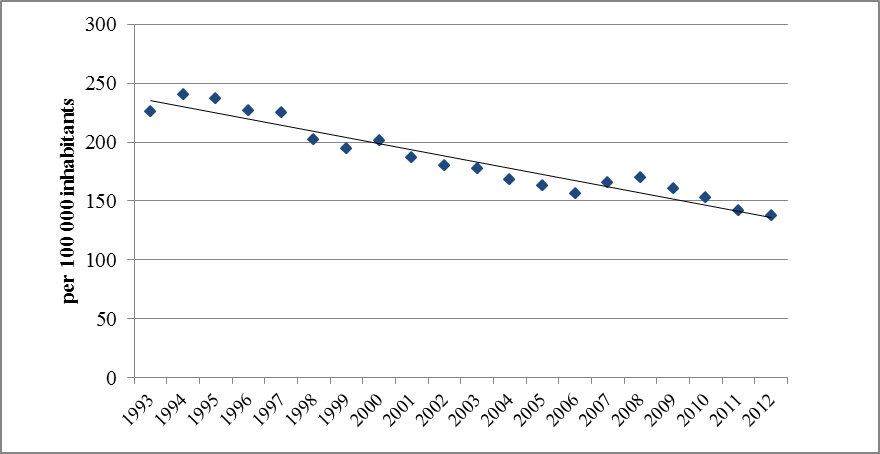


Figure A Age-adjusted mortality from CHD in Slovak population aged 25-74 years between years 1993 – 2012 (both sexes).

Table B CHD mortality rates in Slovakia between 1993-2008, population ages 25-74 years (standardised on European standard population, Δ corresponds to changes in age-adjusted mortality rate)

| **Sex** | **Year** | **Population** | **Deaths** | **Crude Mortality** | **Age-adjusted mortality** | **Δ%** |
| --- | --- | --- | --- | --- | --- | --- |
| **Men** | **1993** | 1467708 | 4507 | 307.08 | 344.60 | -23.5% |
|  | **2008** | 1684159 | 3741 | 222.13 | 263.76 |  |
| **Women** | **1993** | 1589027 | 2341 | 147.32 | 130.90 | -26.1% |
|  | **2008** | 1780062 | 1824 | 102.47 | 96.67 |  |
| **Total** | **1993** | 3056735 | 6848 | 224.03 | 225.99 | -24.7% |
|  | **2008** | 3464221 | 5565 | 160.64 | 170.08 |  |

## 1.2 Heart failure group/deaths

The IMPACT model considers not only CHD deaths (I20-I25) but also CHD related deaths which are mainly represented by heart failure (I50). Many authors of the IMPACT models assumed that all heart failure deaths that are of ischaemic cardiomyopathy origin, are correctly coded with the code I25. However, after consultation with experts we decided to arbitrarily add 50% of deaths coded with I50 to CHD deaths (I20-I25) to reflect the fact that according to the experts often patients are not coded correctly. This is also consistent with the Czech model.^24^ This action did not change the mortality trend significantly (see figure B and table C).


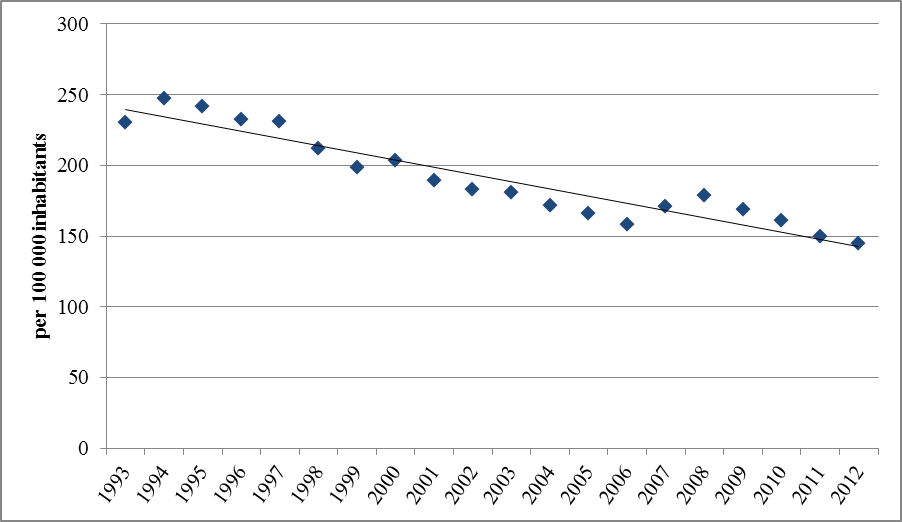


Figure B Age-adjusted mortality from CHD (plus 50% of Heart failure deaths) in Slovak population aged 25-74 years between years 1993 – 2012 (both sexes).

Table C CHD (plus 50% of Heart failure deaths) mortality rates in Slovakia between 1993-2008, population ages 25-74 years (standardised on European Standard Population, Δ corresponds to changes in age-adjusted mortality rate)

| **Sex** | **Year** | **Population** | **Deaths** | **Crude Mortality** | **Age-adjusted mortality** | **Δ%** |
| --- | --- | --- | --- | --- | --- | --- |
| **Males** | **1993** | 1467708 | 4592 | 312.83 | 350.97 | -21% |
|  | **2008** | 1684159 | 3938 | 233.83 | 276.90 |  |
| **Females** | **1993** | 1589027 | 2395 | 150.69 | 134.01 | -24% |
|  | **2008** | 1780062 | 1910 | 107.30 | 101.31 |  |
| **Total** | **1993** | 3056735 | 6987 | 228.54 | 230.57 | -23% |
|  | **2008** | 3464221 | 5848 | 168.81 | 178.62 |  |

## 1.3 Expected and observed number of deaths from CHD

The data sources needed to estimate the expected and observed numbers of deaths from CHD for 2008 are shown in Table A. The expected number of deaths from CHD in 2008 was calculated by multiplying the age-specific mortality rates from CHD in 1993 by the population counts for 2008 in that age-stratum. Summing over all age strata then yielded the *expected* numbers of deaths from CHD. The difference between the numbers of *expected* and *observed* number of deaths from CHD represents the total number of *deaths prevented or postponed* (**DPPs**). The example of DPPs calculation in one age group is provided in the example A.

Example A Calculation of DPPs in one age group.

| In 1993 there were 1448 CHD (and CHD related) deaths among 281310 men aged 55-64 years, which means a mortality rate 663.3/100000. In 2008 in the same age group lived 291013 men. The number of deaths, that were expected , if the 1993 mortality rates had persisted is calculated as follows:  ***mortality rate in 1993 × population in 2008 ≈ 0.006633 × 291013***  ***≈ 1930 expected deaths***  In 2008, however, there were 1254 deaths in this age group. Subtracting the number of observed deaths from the number of expected deaths yields the DPPs in this age group.    ***expected deaths – observed deaths ≈ 1930 - 1254 ≈ 676 DPPs***  This calculation is then repeated for each age group, for men and women. Total number of DPPs is then sum of age specific DPPs. |
| --- |

## **1.4 Calculation of benefits from treatments**

The treatment arm of the model includes the following populations of patients:

- Those hospitalized with an acute myocardial infarction (AMI) during 1993 and 2008.
- Patients hospitalized with unstable angina (UA) during 1993 and 2008.
- Community-dwelling patients who have survived an AMI in the past 10 years.
- Patients who have undergone revascularization procedure (coronary artery bypass grafting [CABG]. or a percutaneous coronary intervention [PTCA] with or without stent for stable angina) within the last year
- Community-dwelling patients with angina pectoris (no revascularization and/or previous MI).
- Patients admitted to hospital with heart failure during 1993 and 2008.
- Community-dwelling patients with heart failure (no hospital admission) in 1993 and 2008.
- Hypertensive individuals without CHD eligible for antihypertensive therapy as primary prevention.
- Hypercholesterolemia subjects without CHD eligible for cholesterol lowering therapy by statins as primary prevention.

The numbers of patients within each of these groups were estimated using statistical administrative databases. As summarized in Table A. For patients with multiple admissions per year with the same diagnosis we used the first admission of a particular fiscal year as the index event.

For each of the patient groups we estimated the number of DPPs that were attributable to various treatments. All treatments of interest are listed in Table D

Table D Clinical efficacy of interventions: relative risk reductions obtained from meta-analyses, and randomized controlled trials

| **Treatments** | **Current relative risk reduction (95% CI)** | **Source paper** |
| --- | --- | --- |
| **Acute myocardial infarction** | | |
| **Thrombolysis** | 31% (95% CI: 14, 45) | Estess,Topol, 2002^32^ |
| **Aspirin** | 15% (95% CI: 11, 19) | **Antithrombotic Trialists' Collaboration, 2002^33^** |
| **Primary angioplasty (STEMI)** | 32% (95% CI: 5, 51) | **Cucherat – Bonnefoy - Tremeu, 2003^34^** |
| **Primary CABG surgery** | 39% (95% CI: 23, 52) | Yusuf et al., 1994^35^ |
| **Beta-blockers** | 4% (95% CI: –8, 15) | Freemantle et al.,1999^36^ |
| **ACE inhibitors** | 7% (95% CI: 2, 11) | Latini et al.,1995^37^ |
| **Community CPR** | 5%–15% (95% CI: 4, 15.3) | Nichol et al.,1999^38^  Rea et al., 2001^39^ |
| **Hospital CPR** | 33% (95% CI: 10, 36) | Tunstall Pedoe et al., 1992^40^  Nadkarni et al., 2006^41^ |
| **Unstable angina** | | |
| **Aspirin** | 15% (95% CI: 11, 19) | **Antithrombotic Trialists' Collaboration, 2002^33^** |
| **Aspirin& Heparin** | 33% (95% CI: –2, 56) | Oler et al., 1996^42^ |
| **Platelet glycoprotein IIB/IIIA inhibitors** | 9% (95% CI: 2, 16) | Boersma et al., 2002^43^ |
| **PCI (NSTEMI)** | 32% (95% CI: 5, 51) | Fox et al., 2005^44^ |
| **Primary CABG surgery** | 39% (95% CI: 19, 60) | Yusuf et al., 1994^35^ |
| **Secondary prevention in CHD patients (following AMI and revascularization)** | | |
| **Aspirin** | 15% (95% CI: 11, 19) | **Antithrombotic Trialists' Collaboration, 2002^33^** |
| **Beta-blockers** | 23% (95% CI: 15, 31) | Freemantle et al.,1999^36^ |
| **ACE inhibitors** | 20% (95% CI: 13, 26) | Flather et al., 2000^45^ |
| **Statins** | 24% (95% CI: 10, 26) | Hulten et al., 2006^46^ |
| **Warfarin** | 22% (95% CI: 13, 31) | Anand - Yusuf, 1999^47^ |
| **Stable angina** | | |
| **CABG surgery years 0-5** | 39% (95% CI: 23, 52) | Yusuf et al., 1994^35^ |
| **CABG surgery years 6-10** | 32% (95% CI: 2, 30) | Yusuf et al., 1994^35^ |
| **Angioplasty in chronic angina, with stents** | No effect | Boden et al., 2007^48^  Cecil et al., 2008^49^ |
| **Aspirin** | 15% (95% CI: 11, 19) | **Antithrombotic Trialists' Collaboration, 2002^33^** |
| **Statins** | 23% (95% CI: 10–26) | Wilt et al., 2004^50^ |
| **Heart failure (hospital, chronic)** | | |
| **ACE Inhibitors** | 20% (95% CI: 13, 26) | Flather et al., 2000^45^ |
| **Beta-blockers** | 35% (95% CI: 26, 43) | Shibata – Flather -Wang, 2001^51^ |
| **Spironolactone** | 31% (95% CI: 18, 42) | Pitt et al., 1999^52^ |
| **Aspirin** | 15% (95% CI: 11, 19) | **Antithrombotic Trialists' Collaboration, 2002^33^** |
| **Statins** | No effect | Kjekshus et al., 2007^53^  Tavazzi et al. ,2008^54^ |
| **Hypertension treatment** | | |
| **Antihypertensives** | 13% (95% CI: 6, 19) | Law -Wald - Morris, 2003^55^ |
| **Therapies for primary prevention of raised cholesterol** | | |
| **Statins** | 35% (95% CI: 11,52) | Pignone – Phillips - Murtlow, 2000^56^ |

The general approach to calculating the number of DPPs from an intervention among a particular patient group was first to stratify by age and sex. Then to multiply the estimated number of patients in the year 2008 by the proportion of these patients receiving a particular treatment, by the 1-year case-fatality rate and by the relative reduction in the case-fatality rate due to the administered treatment. Sources for estimates of efficacy (relative risk reductions) are shown in Table D. Sources for treatment uptakes are shown in Table E. Sources for case-fatality rates are summarised in Table J.

Table E Treatments uptake (weighted average of all age groups) and data sources

|  | **1993** | **Source** | **2008** | **Source** |
| --- | --- | --- | --- | --- |
| ***Acute myocardial infarction*** | | | | |
| **Community CPR** | 0% | - | 0% | Estimate |
| **Hospital CPR** | 1% |  | 4% |  |
| **Thrombolysis** | 33% | AUDIT 1997-1998 | 27% | SLOVAKS 2008 |
| **Primary PTCA (STEMI)** | 0% |  | 59% |  |
| **Primary PTCA (NSTEMI)** | 0% |  | 17% |  |
| **Aspirin** | 73% |  | 94% |  |
| **Beta-blockers** | 45% |  | 82% |  |
| **ACE Inhibitors** | 12% |  | 83% |  |
| **Primary CABG** | 3% |  | 7% |  |
| **Unstable angina** | | | | |
| **Platelet IIB/IIIA Inhibitors** | 0% | 40% of final year (estimate) | 3% | SLOVAKS 2008 |
| **Aspirin** | 37% |  | 92% |  |
| **Aspirin and Heparin** | 33% |  | 83% |  |
| **CABG** | 4% |  | 10% |  |
| **PTCA** | 0% |  | 17% |  |
| **Secondary prevention following AMI** | | | | |
| **Aspirin** | 57% | AUDIT 1997-1998 | 66% | SLOVAKS 2008  SLOVAKS 2011 |
| **Beta blockers** | 40% |  | 58% |  |
| **ACE inhibitors** | 41% |  | 58% |  |
| **Statins** | 0% |  | 60% |  |
| **Warfarin** | 0% |  | 1% |  |
| **Secondary prevention following CABG or PTCA** | | | | |
| **Aspirin** | 58% | AUDIT 1997-1998 | 66% | SLOVAKS 2008  SLOVAKS 2011 |
| **Beta blockers** | 42% |  | 58% |  |
| **ACE inhibitors** | 40% |  | 58% |  |
| **Statins** | 0% |  | 60% |  |
| **Warfarin** | 0% |  | 1% |  |
| **Stable angina in community** | | | | |
| **CABG** | 100% | 100% of those referred | 100% | Z 100% of those referred |
| **Aspirin** | 37% | 60% of final year | 62% | Czech data^24^ |
| **Statins** | 0% | Assumption | 53% |  |
| **Hospital heart failure** | | | | |
| ACE inhibitors | 10% | Expert opinion | 48% | SLOVASeZ 2009 |
| Beta blockers | 0% |  | 60% |  |
| Spironolactone | 0% |  | 49% |  |
| Aspirin | 20% |  | 51% |  |
| **Community heart failure** | | | | |
| ACE inhibitors | 15% | Expert opinion | 73% | Unpublished data from SKS |
| Beta blockers | 0% |  | 71% |  |
| Spironolactone | 0% |  | 36% |  |
| Aspirin | 22% |  | 61% |  |
| **Antihypertensives for primary prevention** | | | | |
| Antihypertensives | 25% | CINDI | 49% | CINDI 2008 & EHES 2011 |
| **Statins for primary prevention** | | | | |
| Statins | 0% | Assumption | 13% | EHES 2011 |

We assumed that compliance (concordance)**,** the proportion of treated patients actually taking therapeutically effective levels of medication, was 100% among hospital patients, 70% among symptomatic community patients and 50% in asymptomatic individuals taking statins or anti-hypertensives for primary prevention.^57, 58^

All these assumptions were tested in subsequent sensitivity analyses.

Example B Calculation of DPPs attributable to evidence based treatment

| For example, in the Slovak Republic in 2008, about 1115 men aged 55–64 were hospitalized with AMI of whom approximately 93% were given aspirin. The underlying case-fatality rate in these men was approximately 5.4%. Aspirin use reduces case-fatality rate by approximately 15%. the deaths prevented or postponed (DPPs) for at least a year were therefore calculated as  ***patients numbers × one year case fatality*** × ***compliance × treatment uptake × relative mortality reduction***  1115 × 0.054 ×1× 0.93 × 0.15 ≈ 8 DPPs  This calculation was then repeated  (a) for men and women in each age group,  (b) for each patient group and treatment,  (c) incorporating Mant and Hicks adjustment for multiple medications (see below),  (d) using maximum and minimum values for each parameter in each group, to generate a sensitivity analysis (see below). |
| --- |

## **1.5 Calculation of influence from risk factors**

The second part of the IMPACT model involves estimating the number of coronary heart disease DPPs related to changes in cardiovascular risk factor levels in the population. The Slovak IMPACT model includes smoking, total cholesterol, systolic blood pressure, body mass index, physical inactivity and diabetes mellitus. Data sources used to calculate the trends in the prevalence (or mean values) of the specific risk factors are shown in Table F.

Table F Risk factors data sources

| **Risk factor** | **1993** | **2008** | **Definition** |
| --- | --- | --- | --- |
| **Current smoking** | CINDI | CINDI | Self-reported status |
| **Systolic blood pressure** | CINDI | CINDI | Average of first and second reading. |
| **Total serum cholesterol** | CINDI | CINDI | Venous blood analysed in biochemical laboratory. |
| **Obesity (BMI)** | CINDI | CINDI | Weight (kg) divided by height squared (m2) for all respondents with valid height and weight measurements. |
| **Diabetes** | GBMD^59^ | GBMD^59^ | - |
| **Physical inactivity** | CINDI | CINDI | Leisure time physical activity stated as: “in my leisure time I read, watch TV or do activities that do not require physical activity” |

Two approaches to calculating DPPs from changes in risk factors were used.

In the **regression approach,** used for systolic blood pressure, total cholesterol, and body mass index (all continuous variables). The numbers of deaths from CHD expected in 2008 were multiplied by the absolute change in risk factor prevalence, and by a regression coefficient quantifying the change in CHD mortality that would result from the change in risk factor level. Natural logarithms were used, as is conventional, in order to best describe the log-linear relationship between changes in risk factor levels and mortality.

Example C Calculation of DPPs attributable to a risk factor change using regression approach

| For example, in 1993, there were 531 CHD deaths among 264,357 women aged 55-64 years. The expected number of deaths in 2008 based on mortality rates from 1993 was 671. Mean total cholesterol in this group then decreased by 0.76 mmol/l mmHg (from 6.46 in 1993 to 5.70 in 2008). The largest meta-analysis reports an estimated age- and sex-specific reduction in mortality of 35% for every 1 mmol/l reduction in total cholesterol, generating a logarithmic coefficient of –0.431. The number of deaths prevented or postponed as a result of this change was then estimated as:  ***(1-e ^(coefficient ×change)^) × deaths expected 2008***  (1-e ^(-0.431^ *^× 0.76)^) × 671≈ 187 DPPs*  This calculation was then repeated  (a) for men and women in each age group, and  (b) using maximum and minimum values in each group, to generate a sensitivity analysis. |
| --- |

Data sources for the coefficients used in these analyses are listed in Table G (a, b, c).

Table G Estimated β coefficients from multiple regression analyses for the relationship between absolute changes in population mean risk factors and percentage changes in coronary heart disease mortality for men and women, stratified by age. Data sources, values and comments.

| **a. Systolic blood pressure** | | **Age group** | | | | |
| --- | --- | --- | --- | --- | --- | --- |
|  |  | **25-44** | **45-54** | **55-64** | **65-74** | **75+** |
| **Men** (hazard ratio per 20 mmHg) | | 0.49 | 0.49 | 0.52 | 0.58 | 0.65 |
| Men (log hazard ratio per 1 mmHg) | | **-0.036** | **-0.035** | **-0.032** | **-0.027** | **-0.021** |
| *Minimum* | | *-0.029* | *-0.028* | *-0.026* | *-0.022* | *-0.017* |
| *Maximum* | | *-0.043* | *-0.042* | *-0.039* | *-0.032* | *-0.025* |
| **Women** (hazard ratio per 20 mmHg) | | 0.40 | 0.40 | 0.49 | 0.52 | 0.59 |
| Women (log hazard ratio per 1 mmHg) | | **-0.046** | **-0.046** | **-0.035** | **-0.032** | **-0.026** |
| *Minimum* | | *-0.037* | *-0.037* | *-0.028* | *-0.026* | *-0.021* |
| *Maximum* | | *-0.055* | *-0.055* | *-0.042* | *-0.039* | *-0.031* |
| Source: Lewington et al., 2002^60^ | | | | | | |
| Units: | Percentage change in CHD mortality per 20 mmHg change in systolic blood pressure | | | | | |
| **Strengths:** | Large dataset, includes US data, adjusted for regression dilution bias, consistent with randomised controlled trials, results stratified by age and sex, with 95% confidence intervals | | | | | |
| **Limitations:** | Some publication bias still possible | | | | | |

| **b. Cholesterol** | **Age group** | | | | | | |
| --- | --- | --- | --- | --- | --- | --- | --- |
|  | **25-44** | | **45-54** | **55-64** | **65-74** | **75-84** | **85+** |
| **Mortality reduction per 1 mmol/l** | | | | | | | |
| Men | 0.55 | | 0.53 | 0.36 | 0.21 | 0.21 | 0.21 |
| Women | 0.57 | | 0.52 | 0.35 | 0.23 | 0.23 | 0.23 |
| **Log coefficient** | | | | | | | |
| **Men** | **-0.799** | | **-0.755** | **-0.446** | **-0.236** | **-0.117** | **-0.083** |
| *Minimum* | *-0.639* | | *-0.604* | *-0.357* | *-0.189* | *-0.093* | *-0.067* |
| *Maximum* | *-0.958* | | *-0.906* | *-0.536* | *-0.283* | *-0.140* | *-0.100* |
| **Women** | **-0.844** | | **-0.734** | **-0.431** | **-0.261** | **-0.174** | **-0.051** |
| *Minimum* | *-0.675* | | *-0.587* | *-0.345* | *-0.209* | *-0.139* | *-0.041* |
| *Maximum* | *-1.013* | | *-0.881* | *-0.517* | *-0.314* | *-0.209* | *-0.062* |
| Source: Lewington et al., 2007^61^ | | | | | | | |
| Units: | | Percentage change in CHD mortality per 1 mmol/l change in total cholesterol | | | | | |
| **Strengths:** | | Includes US data, adjusted for regression dilution bias, includes randomised controlled trials, RCT values consistent with observational data, results stratified by age and sex, with 95% confidence intervals | | | | | |
| **Limitations:** | | Some publication bias still possible | | | | | |

| **c. Body Mass Index (BMI)** | | **Age group** | | | | |
| --- | --- | --- | --- | --- | --- | --- |
|  |  | **<44** | **45-59** | **60-69** | **70-79** | **80+** |
| *James et al. (2004)^62^:* | |  |  |  |  |  |
| Hazard ratio | | 0.89 | 0.91 | 0.95 | 0.96 | 0.97 |
| Risk reduction per 1 kg/m^2^ | | 0.11 | 0.09 | 0.05 | 0.04 | 0.03 |
| Age gradient (45-59 as reference) | | 1.22 | **1.00** | 0.56 | 0.44 | 0.33 |
| *Bogers (2006)^63^:*  *Relative risks, CHD deaths per 5 BMI units (kg/m2)* | |  | **1.16** |  |  |  |
| Relative risks per 1 kg/m2 applying age gradients from James et.al | | 1.04 | 1.03 | 1.02 | 1.01 | 1.01 |
| **Log coefficients** | | **0.0363** | **0.0297** | **0.0165** | **0.0132** | **0.0099** |
| *Minimum* | | *0.0255* | *0.0209* | *0.0116* | *0.0093* | *0.0070* |
| *Maximum* | | *0.0466* | *0.0381* | *0.0212* | *0.0169* | *0.0127* |
| Source: Bogers et al., 2006^63^; James et al. 2004^62^ | | | | | | |
| Units: | Percentage change in CHD mortality per 1 kg/m^2^ change in BMI | | | | | |
| **Strengths:** | Large number of studies included. Adjusted for blood pressure, total cholesterol, and physical activity. 95% confidence intervals included. | | | | | |
| **Limitations:** | Observational data; age gradient applied from James study | | | | | |

The **population-attributable risk fraction (PARF) approach** was used for smoking, physical inactivity and diabetes, being categorical variables. PARF was calculated conventionally as:

**(P** × **(RR**–**1)) / (P** × **(RR**–**1) +1)**

where P is the prevalence of the risk factor and RR is the relative risk for CHD mortality associated with that risk factor. DPPs were then estimated as the expected CHD deaths in 2008 (i.e., if the baseline year mortality persisted) multiplied by the difference in the PARF for 1993 and 2008.

Example D Calculation of DPPs attributable to a risk factor change using PARF method

| For example, the prevalence of smoking among men aged 25-34 years was 31% in 1993 and 26% in 2008. Assuming an RR of 3.33 the PARF was 0.42 in 1993 and 0.37 in 2008. The number of DPPs attributable to the decrease in smoking prevalence from 1993 to 2008 was therefore calculated as:  ***numbers of CHD deaths expected in 2008 × (PARF in 1993 - PARF in 2008)***  37 × (0.42 – 0.37) ≈ 2 DPPs  This calculation was then repeated  (a) for men and women in each age group,  (b) for physical inactivity and smoking,  (c) using maximum and minimum values in each group, to generate a sensitivity analysis. |
| --- |

Sources for the relative risks used in these PARF analyses are listed in Table H. All come from the INTERHEART study^64^, the largest international study to provide *independent* RR values, adjusted for other major risk factors.

Table H Relative risks used in the Slovak IMPACT model for smoking and diabetes for CHD mortality (best, minimum and maximum estimates from the InterHeart Study^a^)

| **Risk factor** | **Both sexes** | | **Men** | | **Women** | |
| --- | --- | --- | --- | --- | --- | --- |
|  | **Young** | **Old** | **≤55 years** | **>55 years** | **≤65 years** | **>65 years** |
| **Smoking** | 3.33 (2.86-3.87) | 0.72 (0.6-0.85) | 3.33 (2.80-3.95) | 2.52 (2.15-2.96) | 4.49 (3.11-6.47) | 2.14 (1.35-3.39) |
| **Exercise** | 0.95 (0.79-1.14) | 0.79 (0.66-0.94) | 1.02 (0.83-1.25)^b^ | 0.79 (0.66-0.96) | 0.74 (0.49-1.10) | 0.75 (0.46-1.22) |
| **Diabetes** | 2.96 (2.40-3.64) | 2.05 (1.71-2.45) | 2.66 (2.04-3.46) | 1.93 (1.58-2.37) | 3.53 (2.49-5.01) | 2.59 (1.78-3.78) |

**^a^**Global InterHeart values were used in the Slovak IMPACT Model

^b^The InterHeart study quoted a value of only 1.02 for exercise in men aged <55 years. This was clearly an outlier. We have therefore assumed a value of 0.77 in line with men and women in the other age groups, and consistent with most other studies.

## **1.6 Other methodological considerations**

Several methodological issues will be discussed below. These include adjusting the relative reduction in case-fatality rate for patients receiving multiple treatments, establishing rules for avoiding double counting individual patients who may fall into more than a single disease category (patient group), treatment overlaps and primary prevention treatments to reduce risk factors.

##

## **Systolic BP and hyperlipidaemia**

In order to separate the DPPs from pharmacological versus non-pharmacological primary prevention of hypertension and hyperlipidaemia, we subtracted the age-gender specific DPPs calculated in the treatment section (i.e. for primary hyperlipidaemia and hypertension patient groups). from the DPPs calculated in the risk factor section.

## **Polypharmacy issues**

Individual CHD patients may take a number of different medications. However. data from randomized clinical trials on efficacy of treatment combinations are sparse. Mant and Hicks suggested a method to estimate case-fatality reduction by polypharmacy^65^.

Example E Mant and Hicks approach example

| If we take the example **of secondary prevention following acute myocardial infarction,** evidence suggests that, for each intervention, the relative reduction in case fatality is approximately: aspirin 15%, beta-blockers 23%, ACE inhibitors 20%, statins 24%, and rehabilitation 26%. The Mant and Hicks approach suggests that, in individual patients receiving all these interventions, case-fatality reduction is very unlikely to be simply additive, i.e., not **108%** (15% + 23% + 20% + 24% + 26%). Instead, having considered the 15% case fatality reduction achieved by aspirin, the next medication, in this case a beta-blocker, can only reduce the **residual** case fatality (1-15%). Likewise, the subsequent addition of an ACE inhibitor can then only decrease the **remaining** case fatality, which will be 1 – [(1– 0.15) × (1–0.23)], and so on.  The Mant and Hicks approach therefore suggests that a **cumulative relative benefit** can be estimated as follows:  ***Relative benefit = 1 – ((1 – relative reduction in case-fatality rate for treatment A) × (1 – relative reduction in case-fatality rate for treatment B) × ... × (1 – relative reduction in case-fatality rate for treatment N).***  For instance, in considering appropriate treatments for AMI survivors, applying relative risk reductions (RRR) for aspirin, beta-blockers, ACE inhibitors, statins, and rehabilitation then gives:  *Relative benefit = 1* – *[(1* – *aspirin RRR) × (1* – *beta-blockers RRR) × (1* – *ACE inhibitors RRR) × (1* – *statins RRR) × (1* – *rehabilitation RRR)]*  = 1 – [(1 – 0.15) *×* (1 – 0.23) *×* (1 – 0.20) *×* (1 – 0.24) *×* (1 – 0.26)]  = 1 – [(0.85) *×* (0.77) *×* (0.80) *×* (0.76) *×* (0.74)]  ≈ 0.71, i.e. a 71% lower case fatality |
| --- |

##

## Potential overlaps between patient groups: avoiding double counting

There are potential overlaps between patient groups. For example. many of the individual patients having CABG surgery have had a previous AMI. some of the AMI survivors develop heart failure within 12 months. and many CHD patients have a history of hypertension. The potential overlaps between CHD patient groups are shown in Table I.

Table I Overlap adjustments in the Slovak IMPACT model

| Following adjustments were made in order to get mutually exclusive groups of patients:   - - - 1. Number of CABG survivors was decreased by 66% assuming 66% CABG patients have a previous AMI, and are therefore already counted as Post MI Secondary prevention.^17, 21^       2. Number of PTCA survivors was decreased by 72% assuming that 50% had an MI and therefore already counted in Post MI 2'prevention group; assuming that 25% go on to CABG and therefore already counted and assuming 25% of PTCA procedures are repeats and therefore individual patient already counted.^17, 21^       3. Numbers of patients with chronic angina without myocardial infarction was decreased by the number of unstable emergency admissions. |
| --- |

## 1.7 Sensitivity analysis

Because of uncertainties surrounding many of the values, a multi-way sensitivity analysis was performed using the analysis of extremes method.^66^ For each model parameter, lower and upper values were assigned using either 95% confidence intervals where available (e.g., therapeutic effectiveness quantified as a relative risk reduction in the relevant meta-analyses), or otherwise plus or minus 20%.

An analysis of extremes was then performed whereby the maximum and minimum feasible values were fed in to the model. By multiplying through, the resulting product then generated maximum and minimum estimates for deaths prevented or postponed.

Example F Sensitivity analysis example

| An example of calculating lower and upper bound estimates for DPPs for treatment with aspirin among men aged 55-64 years who were hospitalized with an AMI is presented here. 95% confidence intervals from the meta-analysis were used for relative mortality reduction; lower and upper bound estimates for the other parameters were calculated as minus or plus 20% (except for treatment uptake that was capped at 99%). Multiplying all the lower-bound estimates yielded the minimum (lower bound) estimate and multiplying the upper-bound estimates yielded the maximum (upper bound) estimate.   \|  \| **Patients numbers** \| **Treatment uptake** \| **Relative mortality reduction** \| **One-year case-fatality** \| **Deaths prevented or postponed** \| \| --- \| --- \| --- \| --- \| --- \| --- \| \| ***a*** \| ***B*** \| ***C*** \| ***d*** \| ***(a × b × c × d)*** \| \| *Best* \| *1115* \| *0.93* \| *0.15* \| *0.054* \| *8* \| \| *Lower extreme* \| *892* \| *0.74* \| *0.12* \| *0.043* \| *3* \| \| *Upper extreme* \| *1338* \| *0.99* \| *0.18* \| *0.065* \| *16* \|     In the Slovak Republic in 2008, about 1115 men aged 55–64 were hospitalized with AMI of whom approximately 93% were given aspirin. The underlying case-fatality rate in these men was approximately 5.4%. Aspirin use reduces case-fatality rate by ≈ 15%. The underlying case-fatality rate in these men was approximately 0.046. The calculated number of deaths prevented or postponed was ≈ 8. A multi-way sensitivity analysis was then performed. The lower and upper bounds for each parameter were estimated using either 95% CI where available, or, failing that, using calculated bounds of plus or minus 20%. |
| --- | --- | --- | --- | --- | --- | --- | --- | --- | --- | --- | --- | --- | --- | --- | --- | --- | --- | --- | --- | --- | --- | --- | --- | --- | --- | --- | --- | --- | --- |

This approach may be described as robust approach for two reasons.

1. Maximum and minimum values for each variable were deliberately forced to provide a wider range rather than a narrower one, e.g. relative mortality reduction ±20% rather than say ±10%.
2. The resulting product, for instance the minimum estimate, was generated by assuming that the lowest feasible values all occurred at the same time, a most unlikely situation.

## 1.8 Treatments effects from the initial year

The IMPACT model aims to explain the causes of the difference between expected and observed deaths from CHD between two points in time. The Slovak version explains the gap between 1993 and 2008. In 1993 there were available some of considered treatments. Therefore we need to subtract their effect from the DPPs in 2008 in order to get net DPPs for 2008 (that are a result of increase in uptakes). Therefore basically second model runs in the IMPACT model for the initial year generating DPPs. The DPPs from 1993 are then subtracted from the DPPs in 2008 resulting in net DPPs in 2008. Only net DPPs are presented in the results.

Table J Case fatality rates for each patient group

|  | **AMI** | **Post AMI** | **Unstable Angina** | **CABG surgery** | **Angioplasty** | **Heart failure**  **hospital** | **Heart failure**  **community** | **Hypertension** | **Hypercholesterolemia** |
| --- | --- | --- | --- | --- | --- | --- | --- | --- | --- |
|  | **30 day** | **One year^a^** | **One year^a^** | **One year^a^** | **One year^a^** | **One year** | **One year** | **One year** | **One year** |
| **Men** | | | | | | | | | |
| **25-34** | *0.011* | 0.008 | 0.016 | 0.003 | 0.003 | 0.034 | 0.011 | 0.000 | 0.000 |
| **35-44** | *0.012* | 0.009 | 0.024 | 0.005 | 0.005 | 0.068 | 0.022 | 0.001 | 0.001 |
| **45-54** | *0.023* | 0.017 | 0.034 | 0.007 | 0.007 | 0.096 | 0.032 | 0.002 | 0.002 |
| **55-64** | *0.054* | 0.034 | 0.056 | 0.012 | 0.012 | 0.140 | 0.045 | 0.006 | 0.006 |
| **65-74** | *0.101* | *0.073* | 0.070 | 0.023 | 0.025 | 0.283 | 0.093 | 0.014 | 0.014 |
| **75-84** | *0.164* | *0.122* | 0.091 | 0.042 | 0.042 | 0.337 | 0.111 | 0.035 | 0.035 |
| **85+** | *0.279* | *0.189* | 0.118 | 0.075 | 0.074 | 0.418 | 0.138 | 0.094 | 0.094 |
| **Women** | | | | | | | | | |
| **25-34** | *0.011* | *0.004* | *0.016* | *0.003* | *0.003* | 0.034 | 0.011 | 0.000 | 0.000 |
| **35-44** | *0.013* | *0.006* | *0.024* | *0.005* | *0.005* | 0.068 | 0.022 | 0.001 | 0.001 |
| **45-54** | *0.026* | *0.010* | *0.034* | *0.007* | *0.007* | 0.096 | 0.032 | 0.001 | 0.001 |
| **55-64** | *0.061* | *0.019* | *0.056* | *0.012* | *0.012* | 0.140 | 0.045 | 0.002 | 0.002 |
| **65-74** | *0.114* | *0.084* | *0.070* | *0.023* | *0.027* | 0.222 | 0.081 | 0.007 | 0.007 |
| **75-84** | *0.167* | *0.116* | *0.091* | *0.042* | *0.039* | 0.289 | 0.094 | 0.021 | 0.021 |
| **85+** | *0.267* | *0.177* | *0.118* | *0.075* | *0.061* | 0.368 | 0.121 | 0.079 | 0.079 |
| **Source** | Medicare | Medicare | van Domburg, van Miltenburg-van Zijl et al. 1998^67^ | Medicare | Medicare | Medicare | Medicare | NHANES & Vital Statistics | |

^a^ excluding heart failure patients (already considered within heart failure groups)

Table K Effects of treatments methods to the CHD mortality decrease between the years 1993-2008 in the population of 25-74 years old Slovaks (extended)

|  | **Patients eligible** | | **Uptake^a^** | | **RRR** | **CFR** | **Deaths prevented or postponed^b^** | | | **Deaths prevented or postponed (%)** | | |
| --- | --- | --- | --- | --- | --- | --- | --- | --- | --- | --- | --- | --- |
|  | *1993* | *2008* | *1993* | *2008* |  |  | *Best* | *Min.* | *Max.* | *Best* | *Min.* | *Max.* |
| **Initial treatments of AMI** |  |  |  |  |  |  | **75** | **40** | **140** | **4.1** | **2.1** | **7.6** |
| Community CPR | 2015 | 1437 | 0% | 0% | 0.05 | 0.066 | 0 | 0 | 0 | 0.0 | 0.0 | 0.0 |
| Hospital CPR | 6106 | 4354 | 1% | 4% | 0.32 | 0.066 | 30 | 20 | 50 | 1.6 | 1.0 | 2.8 |
| Thrombolysis | 6106 | 4354 | 33% | 27% | 0.27 | 0.066 | -15 | -5 | -30 | -0.8 | -0.3 | -1.7 |
| Aspirin | 6106 | 4354 | 73% | 94% | 0.15 | 0.066 | -10 | -5 | -30 | -0.6 | -0.2 | -1.6 |
| β-blockers | 6106 | 4354 | 45% | 82% | 0.04 | 0.066 | 0 | 0 | 5 | 0.1 | 0.0 | 0.1 |
| ACEi | 6106 | 4354 | 12% | 83% | 0.07 | 0.066 | 10 | 5 | 20 | 0.6 | 0.2 | 1.2 |
| PTCA (STEMI) | 6106 | 4354 | 0% | 59% | 0.32 | 0.066 | 45 | 20 | 90 | 2.4 | 1.0 | 5.0 |
| PTCA (NSTEMI) | 6106 | 4354 | 0% | 17% | 0.32 | 0.066 | 10 | 5 | 24 | 0.7 | 0.3 | 1.4 |
| CABG | 6106 | 4354 | 3% | 7% | 0.39 | 0.066 | 5 | 0 | 5 | 0.1 | 0.0 | 0.3 |
| **Unstable angina** |  |  |  |  |  |  | **90** | **40** | **180** | **4.9** | **2.0** | **9.8** |
| Aspirin & Heparin | 4393 | 7326 | 33% | 83% | 0.33 | 0.056 | 45 | 20 | 95 | 2.5 | 1.0 | 5.2 |
| Aspirin alone | 4393 | 7326 | 37% | 92% | 0.15 | 0.056 | 25 | 10 | 40 | 1.3 | 0.5 | 2.3 |
| PG IIb/IIIa | 4393 | 7326 | 0% | 3% | 0.09 | 0.056 | 0 | 0 | 0 | 0.0 | 0.0 | 0.1 |
| CABG | 4393 | 7326 | 4% | 10% | 0.39 | 0.056 | 5 | 5 | 15 | 0.4 | 0.2 | 0.8 |
| PTCA | 4393 | 7326 | 0% | 17% | 0.32 | 0.056 | 15 | 5 | 30 | 0.7 | 0.3 | 1.5 |
| **Secondary prevention following AMI** |  |  |  |  |  |  | **130** | **35** | **365** | **7.0** | **2.0** | **20.0** |
| Aspirin | 32885 | 37613 | 57% | 66% | 0.15 | 0.040 | -5 | 0 | -10 | -0.2 | -0.1 | -0.6 |
| β-blockers | 32885 | 37613 | 40% | 58% | 0.23 | 0.040 | 30 | 10 | 70 | 1.5 | 0.5 | 3.8 |
| ACEi | 32885 | 37613 | 41% | 58% | 0.20 | 0.040 | 10 | 5 | 25 | 0.5 | 0.2 | 1.3 |
| Statins | 32885 | 37613 | 0% | 60% | 0.24 | 0.040 | 95 | 25 | 275 | 5.1 | 1.3 | 15.2 |
| Warfarin | 32885 | 37613 | 0% | 1% | 0.22 | 0.040 | 0 | 0 | 5 | 0.1 | 0.0 | 0.3 |
| **Secondary prevention following CABG and PTCA** |  |  |  |  |  |  | **20** | **5** | **55** | **1,2** | **0,4** | **3,0** |
| Aspirin | 129 | 9627 | 58% | 66% | 0.15 | 0.013 | 5 | 0 | 10 | 0.2 | 0.1 | 0.6 |
| β-blockers | 129 | 9627 | 42% | 58% | 0.23 | 0.013 | 5 | 0 | 15 | 0.3 | 0.1 | 0.8 |
| ACEi | 129 | 9627 | 40% | 58% | 0.20 | 0.013 | 5 | 0 | 15 | 0.3 | 0.1 | 0.7 |
| Statins | 129 | 9627 | 0% | 60% | 0.24 | 0.013 | 5 | 0 | 15 | 0.4 | 0.1 | 0.9 |
| Warfarin | 129 | 9627 | 0% | 1% | 0.22 | 0.013 | 0 | 0 | 0 | 0.0 | 0.0 | 0.0 |
| **Stable angina** |  |  |  |  |  |  | **80** | **25** | **205** | **4.4** | **1.5** | **11.2** |
| CABG | 82 | 9318 | 100% | 100% | 0.35 | 0.013 | 20 | 10 | 35 | 1.1 | 0.6 | 2.0 |
| Aspirin | 14133 | 13150 | 37% | 62% | 0.15 | 0.057 | 15 | 5 | 35 | 0.7 | 0.2 | 1.8 |
| Statins | 14133 | 13150 | 0% | 53% | 0.23 | 0.057 | 45 | 10 | 135 | 2.5 | 0.7 | 7.4 |
| **Hospital heart failure** |  |  |  |  |  |  | **100** | **30** | **275** | **5.5** | **1.6** | **15.1** |
| ACEi | 536 | 2292 | 10% | 48% | 0.20 | 0.196 | 15 | 5 | 45 | 0.9 | 0.2 | 2.6 |
| β-blockers | 536 | 2292 | 0% | 60% | 0.35 | 0.196 | 35 | 10 | 110 | 2.0 | 0.5 | 5.9 |
| Spironolactone | 536 | 2292 | 0% | 49% | 0.30 | 0.196 | 35 | 10 | 85 | 1.8 | 0.6 | 4.6 |
| Aspirin | 536 | 2292 | 20% | 51% | 0.15 | 0.196 | 15 | 5 | 40 | 0.8 | 0.3 | 2.1 |
| **Community heart failure** |  |  |  |  |  |  | **110** | **35** | **285** | **6.1** | **1.9** | **15.6** |
| ACEi | 6294 | 7460 | 15% | 73% | 0.20 | 0.065 | 20 | 5 | 55 | 1.0 | 0.3 | 3.0 |
| β-blockers | 6294 | 7460 | 0% | 71% | 0.35 | 0.065 | 55 | 20 | 135 | 3.0 | 1.0 | 7.4 |
| Spironolactone | 6294 | 7460 | 0% | 36% | 0.31 | 0.065 | 25 | 10 | 65 | 1.4 | 0.5 | 3.5 |
| Aspirin | 6294 | 7460 | 22% | 61% | 0.15 | 0.065 | 10 | 5 | 30 | 0.7 | 0.2 | 1.7 |
| **Statins (primary prevention)** | 1980075 | 1859184 | 0% | 13% | 0.35 | 0.003 | **165** | **55** | **405** | **9.0** | **2.9** | **22.3** |
| **Antihypertensives (primary prevention)** | 1019913 | 1382615 | 25% | 49% | 0.13 | 0.004 | **145** | **25** | **205** | **8.0** | **1.3** | **11.3** |
| **Total treatments** |  |  |  |  |  |  | **915** | **-** | **-** | **50.2%** | **-** | **-** |

^a^ weighted average of all age groups and both sexes; ^b^ net effect after subtracting the 1993 effect; RRR – relative risk reduction; CFR – case fatality rate

The absolute numbers of DPPs were rounded to the nearest multiple of 5 (e.g. 6 became 5), therefore little inaccuracies may occur.

Figure C Contribution of treatments and risk factors changes to the CHD mortality decrease in the population of 25-74 years old Slovaks. Diamonds represent the best estimate; lines are extremes of sensitivity analysis.

Figure D Comparison of observed and explained number of DPPs according to the Slovak IMPACT model.

1. **LIST OF ASSUMPTIONS**

Because there were deficient data we had to make explicit assumptions in order to supplement these missing inputs. Below is the list of all assumptions made within the Slovak IMPACT model.

| 1. **Patients numbers**    1. ***Numbers of patients admitted yearly***       1. The numbers of patients admitted yearly into hospitals (I20; I21&I22; I50) are numbers of individuals. The data come from NCZI. The data are not available for years 1993 and 1994. We used a correction factor in order to estimate this numbers from numbers of beds used by patients with I20; I21&I22 and I50. For each age group we calculated a correction factor based on 1995,1996, 1997 as follows: 1-(number of individuals/number of beds) = the proportion by which is the number of beds higher than number of individuals. The correction factor is a median of three years. We have chosen median, because it happens that the difference between numbers of bed and numbers of individuals was zero. Since average is sensitive for extreme values, we have decided to use median of three years. These correction factors we then used to calculate the number of individuals in 1993 and 1994. Individuals (estimate) = Nr. of beds - (Nr of beds*correction factors median).       2. The numbers of patients admitted into hospital with 428; I50 is decreased by 50% as we assume that 50% of HF is a result of CHD.       3. The number of patients eligible for community CPR is approx. 1/3 of those admitted into hospital (1993 and 2008).       4. All the patients admitted into hospital with AIM are eligible for in-hospital CPR (1993 and 2008).    2. ***Numbers of patients treated yearly***       1. The number of patients treated yearly with PTCA is an estimation based on Slovak total data of PTCAs (unpublished data from NÚSCH) and data about patient age & sex split in NÚSCH. The estimation process was as follows: From databases of NÚSCH all patients with diagnosis I20 - I25 and PTCA were selected and stratified according to age and sex. The proportions of men & women and individual age categories were calculated. Since the databases of NÚSCH are available only from 1998 - 2008 we had to make estimation for ratios in 1993 - 1997. For this purpose we used an average of years 1998, 1999 and 2000.We have available the total numbers of PTCA cases in Slovakia during 1993 - 2008. The data for 1995 and 2007 are missing. They were estimated as midpoint between two most close years. The Slovak numbers of PTCA were then split firstly by gender and then by age using NÚSCH age & sex split.       2. The numbers of patients treated with CABG is an estimation based on data from NÚSCH and NCZI (some missing years had to be estimate using extrapolation). Sex and age split has been done using PTCA ratios from NÚSCH.    3. ***Numbers of patients in the community***        1. The number of patients eligible for 2'prevention following AMI in 2008 is calculated from the data about patients admitted into hospital with I21&I22 (cumulative probability of surviving - last 10 years).       2. The number of patients eligible for 2'prevention following AMI in 1993 is calculated as follows: from the above mentioned data about patient eligible for 2'prevention following AMI in 2008 we calculated the prevalence of patients surviving AMI. We then applied the same prevalence for calculation the number of patients eligible for 2'prevention following AMI in 1993 assuming the prevalence remained unchanged.       3. Numbers of patients eligible for 2'prevention following CABG and PTCA in 2008 were estimated as cumulative probability of surviving (last 10 years).       4. Numbers of patients eligible for 2'prevention following CABG and PTCA in 1993 were estimated from cumulative probability of surviving the CABG and PTCA in 1994, 1995 and 1996. Using exponential extrapolation we then estimated the number in 1993.       5. The number of people living with stable angina pectoris in 2008 is estimated from EHIS 2009. The prevalence of self-reported prevalence of patients with angina but without myocardial infarction is calculated (in order to adjust for overlaps) and is decreased by 50% to reduce overestimation from self-reporting. The rationale for this are for example the results of the INTERGENE study.       6. The number of people living with stable angina pectoris in 1993 is estimated using the same prevalence as in final year.       7. The number of people living in the community with chronic heart failure is calculated from the information of NCZI. The number of people followed up in Cardiologist´s practice in 2008^68^. We increased this number by 10% in order to avoid underreporting.       8. The number of people living in the community with chronic heart failure is calculated using the prevalence from 2008 decreased by 10% assuming the prevalence increased during the observed period.       9. The number of patients eligible for antihypertensive and statins therapy as 1' prevention is calculated based on prevalence of people with SBP ≥140 mmHg or DBP ≥90 mmHg and also those reporting having hypertension and total cholesterol ≥5.0 mmol/l and those reporting having hypercholesterolemia, respectively, without CHD and stroke in anamnesis based on CINDI 1993 and 2008 survey. |
| --- |
| 1. **Risk factors prevalence and mean values**    1. There are no representative data about risk factor prevalence and mean values in Slovakia in the age group 65-74. We had to estimate these values based on previous age groups using linear, polynomic and exponential extrapolations or assuming the prevalence is same as the last available age group (55-64, case of physical inactivity).    2. Prevalence of smoking in the oldest age group was estimated as follows: We used prevalence of regular smokers from CINDI 1993 - 2008. We subsequently used daily smokers prevalence from EHIS 2009 (not counting occasional smokers) and calculated by how much the 65-74 age group differs from the 55-64 age group, i.e. we calculated a correction factors for males and females respectively. We used these correction factors to supplement the last needed age group in data coming from CINDI. We used the same correction factors for 2008 and 1993.    3. There is very limited and possibly not reliable data about self-reported diabetes prevalence in 1993 coming from CINDI. For final year there are more surveys (CINDI 2008, EHIS 2009 and EHES 2011) with different estimates of diabetes prevalence. Given this fact and the fact that the criteria for diagnosing diabetes changed between base and final years, we decided to use data from Global Burden of Metabolic Diseases Project.^59^    4. Mean systolic blood pressure trends are very unstable. From four CINDI screenings it can be seen, that the blood pressure increased/decreased within the observed years. In order to eliminate these fluctuations we used linear extrapolation in order to input data about systolic blood pressure. We conducted linear extrapolation in each age group and the obtained values are now inputs in the model. |
| 1. **Treatments uptake**    1. We assume that community CPR uptake in 1993 and 2008 is very low (zero).    2. The uptake of in-hospital CPR in 1993 was 1% (based on Polish assumptions^21^). The uptake of in-hospital CPR in 2008 is the same as it was in Poland (4%)^21^.    3. For treatment uptake in initial treatments for AMI and 2'prevention following AMI in 1993 we used the AUDIT study conducted between years 1997 – 1998. All the data from the AUDIT study are decreased by 10% in order to reflect the difference between the time of conducting the AUDIT study and our base year.    4. For PTCA in acute phase we assume 0, given the fact an expert stated there were 125 PTCA in 1993 but no PTCA during acute phase of AMI (all of them were elective).    5. For the same categories and unstable angina in final year we used data from Slovak Registry of ACS – SLOVAKS 2008.    6. Unstable angina treatments in 1993 – assume 40% of final year.    7. 2'prevention following AMI & following CABG and PTCA in 1993 – data from AUDIT decreased by 30% reflecting the different years and the fact that this data come from the time at discharge and we assume them go down even more. Assume the same uptake in both patient groups.    8. 2'prevention following AMI & following CABG and PTCA in 2008 – SLOVAKS 2008 (acute phase) decreased by 30%. Assume the same uptake in both patients groups.    9. For acute heart failure in 2008 we used uptakes coming from the SLOVSeZ 2009 study.    10. For acute heart failure treatments uptake in 1993 expert opinions were used.    11. For chronic heart failure in 2008 we used uptakes from unpublished data from year 201.    12. For uptakes of treatment of stable angina we used Czech data^24^ in 2008 (decreased by 30%) and assumption of 60% uptake of the final year in 1993.    13. We assume no statins were in use in 1993.    14. For 2008 data from EHES 2011 (decreased by 10%) were used. We selected all persons without CHD, angina pectoris or stroke and eligible for statin treatments (i.e. cholesterol ≥ 5mmol/l or self-reported hypercholesterolemia) and selected among them all those who reported receiving cholesterol lowering medicaments (assuming cholesterol lowering medicaments = statins).    15. Antihypertensive treatment is calculated from CINDI survey 1993 for initial year. Based on expert opinion we increased this number by 50% in order to be closer to reality. The final year uptake is an average of CINDI 2008 and EHES 2011. We used following approach: We selected people eligible for AHT therapy. In this sample we then selected the people who reported that their blood pressure is not too high currently given the fact they are taking medicaments. |

1. **REFERENCES**

1. Cagán S, Trnovec T. An audit of cardiovascular diseases. Audit kardiovaskulárnych chorôb. 1998 Bratisl lek Listy; 99: 131-137.

2. Jurkovičová O, Cagáň S, Wimmerová S, Besedová I, Trnovec T. Management of patients with acute myocardial infarction at hospital discharge. Manažment chorých s akútnym infarktom myokardu pri prepustení z nemocnice. 2003 Cardiology; 12: 174-183.

3. Jurkovičová O, Cagáň S, Wimmerová S, Besedová I, Trnovec T. Podporná liečba akútneho infarktu myokardu vo včasnom období hospitalizácie I. Liečba kyselinou acetylosalicylovou, betablokátormi a inhibítormi enzímu konvertujúceho angiotenzín. Cardiology 2002; 11.

4. Kovář F, Studenčan M, Hricák V, Kurray P, Murín J, Kamenský G et al. Management of patients with acute coronary syndrome without ST-segment elevation. Analysis of the SLOVAKS registry during 2008 year. Manažment pacientov s akútnym koronárnym syndrómom bez elevácií segmentov ST. Analýza údajov registra SLOVAKS z roku 2008. Cardiology sk 2010; 19: 181-191.

5. Studenčan M, Kovář F, Hricák V, Kurray P, Goncalvesova E, Kamensky G. Aktuálne trendy v starostlivosti o pacientov so STEMI v Slovenskej republike. Analýza výsledkov registra SLOVAKS-2 z roku 2011. Cardiology Lett 2013; 22: 115-124.

6. Gonçalvesová E, Varga I, Lesný P, Líška B, Luknár M, Solík P. Charakteristiky a osud pacientov s akútnym srdcovým zlyhávaním v aktuálnej klinickej praxi. Vnitř lék 2010; 56: 845-853.

7. Avdičová M, Francisciová K, Ďateľová M. Monitorovanie rizikových faktorov chronických chorôb v SR Banská Bystrica: Regionálny úrad verejného zdravotníctva so sídlom v Banskej Bystrici; 2012.

8. Gerhardtová A. EHIS 2009 – Európske zisťovanie o zdraví 2009. Štatistický úrad Slovenskej republiky; 2011.

9. Psota M, Capewell S, O'Flaherty M, Goncalvesova E. Príčiny zmien v úmrtnosti na ischemickú chorobu srdca podľa modelu IMPACT: systematický prehľad / The causes of changes in coronary heart disease mortality rates using the IMPACT model: a systematic review. Cardiology Lett 2013; 22: 449-458.

10. Capewell S, Morrison CE, McMurray JJ. Contribution of modern cardiovascular treatment and risk factor changes to the decline in coronary heart disease mortality in Scotland between 1975 and 1994. Heart 1999; 81: 380-386.

11. Capewell S, Beaglehole R, Seddon M, McMurray J. Explanation for the decline in coronary heart disease mortality rates in Auckland, New Zealand, between 1982 and 1993. Circulation 2000; 102: 1511-1516.

12. Unal B, Critchley JA, Capewell S. Explaining the decline in coronary heart disease mortality in England and Wales between 1981 and 2000. Circulation 2004; 109: 1101-1107.

13. Critchley J, Liu J, Zhao D, Wei W, Capewell S. Explaining the increase in coronary heart disease mortality in Beijing between 1984 and 1999. Circulation 2004; 110: 1236-1244.

14. Laatikainen T, Critchley J, Vartiainen E, Salomaa V, Ketonen M, Capewell S. Explaining the decline in coronary heart disease mortality in Finland between 1982 and 1997. Am J Epidemiol 2005; 162: 764-773.

15. Bennett K, Kabir Z, Unal B, Shelley E, Critchley J, Perry I et al. Explaining the recent decrease in coronary heart disease mortality rates in Ireland, 1985-2000. J Epidemiol Community Health 2006; 60: 322-327.

16. Unal B, Capewell S, Critchley J. Coronary Heart Disease Policy Models: a systematic review BMC Public Health 2006; 6: 213.

17. Ford ES, Ajani UA, Croft JB, Critchley JA, Labarthe DR, Kottke TE et al. Explaining the decrease in US deaths from coronary disease, 1980-2000. N Engl J Med 2007; 356: 2388-2398.

18. Bjorck L, Rosengren A, Bennett K, Lappas G, Capewell S. Modelling the decreasing coronary heart disease mortality in Sweden between 1986 and 2002. Eur Heart J 2009; 30: 1046-1056.

19. Aspelund T, Gudnason V, Magnusdottir BT, Andersen K, Sigurdsson G, Thorsson B, et al. Analysing the Large Decline in Coronary Heart Disease Mortality in the Icelandic Population Aged 25-74 between the Years 1981 and 2006. PLoS One 2010; 5.

20. Palmieri L, Bennett K, Giampaoli S, Capewell S. Explaining the Decrease in Coronary Heart Disease Mortality in Italy Between 1980 and 2000. Am J Public Health 2010; 100: 684-692.

21. Bandosz P, O'Flaherty M, Drygas W, Rutkowski M, Koziarek J, Wyrzykowski B et al. Decline in mortality from coronary heart disease in Poland after socioeconomic transformation: modelling study. BMJ 2012; 344: d8136-d8136.

22. Rastam S, Ali RAL, Maziak W, Mzayek F, Fouad FM, O'Flaherty M et al. Explaining the increase in coronary heart disease mortality in Syria between 1996 and 2006. BMC Public Health 2012; 12: 754-754.

23. Bajekal M, Scholes S, Love H, Hawkins N, O'Flaherty M, Raine R et al. Analysing Recent Socioeconomic Trends in Coronary Heart Disease Mortality in England, 2000-2007: A Population Modelling Study. PLoS Medicine 2012; 9: 1237-1237.

24. Bruthans J, Cífková R, Lánská V, O'Flaherty M, Critchley JA, Holub J et al. Explaining the decline in coronary heart disease mortality in the Czech Republic between 1985 and 2007. Eur J Prev Cardiol 2014; 21: 829-839.

25. Abu-Rmeileh NME, Shoaibi A, Husseini A, O'Flaherty M, Capewell S. Analysing falls in coronary heart disease mortality in the West Bank between 1998 and 2009. BMJ Open 2012; 2.

26. Pereira M, Azevedo A, Lunet N, Carreira H, O’Flaherty M, Capewell S, Bennett K. Explaining the Decline in Coronary Heart Disease Mortality in Portugal Between 1995 and 2008. (Circ Cardiovasc Qual Outcomes 2013; 6.

27. Unal B, Sozmen K, Arik H, Gerceklioglu G, Altun D, Simsek H et al. Explaining the decline in coronary heart disease mortality in Turkey between 1995 and 2008. BMC Public Health 2013; 13: 1135.

28. Hotchkiss JW, Davies CA, Dundas R, Hawkins N, Jhund PS, Scholes S et al. Explaining trends in Scottish coronary heart disease mortality between 2000 and 2010 using IMPACT_SEC_ model: retrospective analysis using routine data. BMJ 2014; 348.

29. Saidi O, Ben Mansour N, O’Flaherty M, Capewell S, Critchley JA, Romdhane HB. Analyzing Recent Coronary Heart Disease Mortality Trends in Tunisia between 1997 and 2009. Plos One 2013; 8: e63202.

30. Wijeysundera HC, Machado M, Farahati F, Wang XS, Witteman W, van der Velde G et al. Association of Temporal Trends in Risk Factors and Treatment Uptake With Coronary Heart Disease Mortality, 1994-2005. JAMA 2010; 303: 1841-1847.

31. Waterhouse JAH, Muir C.S., Correa P, Powell J. Cancer in five continents. Lyon: IARC; 1976.

32. Estess JM, Topol EJ. Fibrinolytic treatment for elderly patients with acute myocardial infarction. Heart 2002; 87(4): 308-311.

33. Collaborative meta-analysis of randomised trials of antiplatelet therapy for prevention of death, myocardial infarction, and stroke in high risk patients. BMJ 2002; 324: 71-86.

34. Cucherat M, Bonnefoy E, Tremeau G. Primary angioplasty versus intravenous thrombolysis for acute myocardial infarction. Cochrane database of systematic reviews (Online : Update Software) 2000 (2).

35. Yusuf S, Zucker D, Peduzzi P, Fisher LD, Takaro T, Kennedy JW et al. Effect of coronary artery bypass graft surgery on survival: Overview of 10-year results from randomised trials by the Coronary Artery Bypass Graft Surgery Trialists Collaboration. Lancet 1994; 344: 563-570.

36. Freemantle N, Cleland J, Young P, Mason J, Harrison J. β blockade after myocardial infarction: Systematic review and meta regression analysis. BMJ 1999; 318: 1730-1737.

37. Latini R, Maggioni AP, Flather M, Sleight P, Tognoni G. ACE inhibitor use in patients with myocardial infarction: Summary of evidence from clinical trials. Circulation 1995; 92: 3132-3137.

38. Nichol G, Stiell IG, Hebert P, Wells GA, Vandemheen K, Laupacis A. What Is the Quality of Life for Survivors of Cardiac Arrest? A Prospective Study. Academic Emergency Medicine 1999; 6: 95-102.

39. Rea TD, Eisenberg MS, Culley LL, Becker L. Dispatcher-assisted cardiopulmonary resuscitation and survival in cardiac arrest. Circulation 2001; 104: 2513-2516.

40. Tunstall Pedoe H, Bailey L, Chamberlain D, Marsden A, Ward MJ, Zideman D. Survey of 3765 cardiopulmonary resuscitations in British hospitals (the BRESUS Study): methods and overall results. BMJ 1992; 304: 1347-1351.

41. Nadkarni VM, Larkin G, Peberdy M, et al. FIrst documented rhythm and clinical outcome from in-hospital cardiac arrest among children and adults. JAMA 2006; 295: 50-57.

42. Oler A, Whooley MA, Oler J, Grady D. Adding heparin to aspirin reduces the incidence of myocardial infarction and death in patients with unstable angina: A meta-analysis. JAMA 1996; 276: 811-815.

43. Boersma E, Harrington RA, Moliterno DJ, White H, Théroux P, Van de Werf F et al. Platelet glycoprotein IIb/IIIa inhibitors in acute coronary syndromes: a meta-analysis of all major randomised clinical trials. The Lancet 2002; 359: 189-198.

44. Fox KAA, Poole-Wilson P, Clayton TC, Henderson RA, Shaw TRD, Wheatley DJ et al. 5-year outcome of an interventional strategy in non-ST-elevation acute coronary syndrome: the British Heart Foundation RITA 3 randomised trial. The Lancet 2005; 366: 914-920.

45. Flather MD, Yusuf S, Køber L, Pfeffer M, Hall A, Murray G et al. Long-term ACE-inhibitor therapy in patients with heart failure or left-ventricular dysfunction: a systematic overview of data from individual patients. The Lancet 2000; 355: 1575-1581.

46. Hulten E, Jackson JL, Douglas K, George S, Villines TC. The effect of early, intensive statin therapy on acute coronary syndrome: A meta-analysis of randomized controlled trials. Archives of Internal Medicine 2006; 166: 1814-1821.

47. Anand SS, Yusuf S. Oral anticoagulant therapy in patients with coronary artery disease: A meta-analysis. JAMA 1999; 282: 2058-2067.

48. Boden WE, O'Rourke RA, Teo KK, Hartigan PM, Maron DJ, Kostuk WJ et al. Optimal Medical Therapy with or without PCI for Stable Coronary Disease. N Engl J Med 2007; 356: 1503-1516.

49. Cecil WT, Kasteridis P, Barnes Jr JW, Mathis RS, Patric K, Martin S. A meta-analysis update: Percutaneous coronary interventions. American Journal of Managed Care 2008; 14: 521-528.

50. Wilt TJ, Bloomfield HE, MacDonald R, Nelson D, Rutks I, Ho M, et al. Effectiveness of statin therapy in adults with coronary heart disease. Archives of Internal Medicine 2004; 164: 1427-1436.

51. Shibata MC, Flather MD, Wang D. Systematic review of the impact of beta blockers on mortality and hospital admissions in heart failure. European Journal of Heart Failure 2001; 3: 351-357.

52. Pitt B, Zannad F, Remme WJ, Cody R, Castaigne A, Perez A et al. The Effect of Spironolactone on Morbidity and Mortality in Patients with Severe Heart Failure. N Engl J Med Medicine 1999; 341: 709-717.

53. Kjekshus J, Apetrei E, Barrios V, Böhm M, Cleland JGF, Cornel JH et al. Rosuvastatin in Older Patients with Systolic Heart Failure. N Engl J Med 2007; 357: 2248-2261.

54. Tavazzi L, Maggioni AP, Marchioli R, Barlera S, Franzosi MG, Latini R et al. Effect of rosuvastatin in patients with chronic heart failure (the GISSI-HF trial): a randomised, double-blind, placebo-controlled trial. The Lancet; 372: 1231-1239.

55. Law M, Wald N, Morris J. Lowering blood pressure to prevent myocardial infarction and stroke: a new preventive strategy. Health Technology Assessment 2003; 7: 106.

56. Pignone M, Phillips C, Mulrow C. Use of lipid lowering drugs for primary prevention of coronary heart disease: meta-analysis of randomised trials. BMJ 2000; 321: 983.

57. Nichol MB, Venturini F, Sung JCY. A critical evaluation of the methodology of the literature on medication compliance. Annals of Pharmacotherapy 1999; 33: 531-540.

58. Butler J, Arbogast PG, BeLue R, Daugherty J, Jain MK, Ray WA et al. Outpatient adherence to beta-blocker therapy after acute myocardial infarction. Journal of the American College of Cardiology 2002; 40: 1589-1595.

59. Danaei G, Finucane M, Lu Y, Singh G, Cowan M, Paciorek C, Lin J, Farzadfar F, Khang Y, Stevens G. National, regional, and global trends in fasting plasma glucose and diabetes prevalence since 1980: systematic analysis of health examination surveys and epidemiological studies with 370 country-years and 2.7 million participants. Lancet 2011; 378: 31-40.

60. Lewington S, Clarke R, Qizilbash N, Peto R, Collins R. Age-specific relevance of usual blood pressure to vascular mortality: a meta-analysis of individual data for one million adults in 61 prospective studies. The Lancet 2002; 360: 1903-1913.

61. Lewington S, Whitlock G, Clarke R, Sherliker P, Emberson J. Blood cholesterol and vascular mortality by age, sex, and blood pressure: a meta-analysis of individual data from 61 prospective studies with 55 000 vascular deaths. The Lancet 2007; 370: 1829-1839.

62. James W, Jackson-Leach R, Mhurchu C, Kalamara E, Shayeghi M. Overweight and obesity (high body mass index). . In: Ezatti M, Lopez AD, Rodgers A, Murray CJL, (eds). Comparative quantification of risk. Global and regional burden of disease attributable to selected major risk factors. . Geneva: World Health Organisation; 2004, 497-596.

63 Bogers R, Hoogenveen R, Boshuizen H, Woodward M, Knekt P. Overweight and obesity increase the risk of coronary heart disease: A pooled analysis of 30 prospective studies. European Journal of Epidemiology 2006; 21(Suppl).

64. Yusuf S, Hawken S, Ounpuu S, Dans T, Avezum A, Lanas F et al. Effect of potentially modifiable risk factors associated with myocardial infarction in 52 countries (the INTERHEART study): case-control study. Lancet 2004; 364: 937-952.

65. Mant J, Hicks N. Detecting differences in quality of care: the sensitivity of measures of process and outcome in treating acute myocardial infarction. BMJ (Clinical Research Ed.) 1995; 311: 793-796.

66. Briggs A, Sculpher MJ, Buxton MJ. Uncertainty in the economic evaluation of health care technologies: the role of sensitive analysis. Health Economics 1994; 3: 95-104.

67. van Domburg RT, van Miltenburg-van Zijl AJ, Veerhoek RJ, Simoons ML. Unstable Angina: Good Long-Term Outcome After a Complicated Early Course. Journal of the American College of Cardiology 1998; 31: 1534-1539.

68. NCZI. Činnosť v kardiologických ambulanciách v SR 2008. Bratislava: Národné Centrum Zdravotníckych Informácií; 2009.

1. Reduced by 50% assuming that 50% of HF is caused by CHD. [↑](#footnote-ref-1)
2. Reduced by 50% assuming that 50% of HF is caused by CHD. [↑](#footnote-ref-2)
